# A Systematic Review and Meta-Analysis of Cerebrospinal Fluid Amyloid and Tau Levels Identifies Mild Cognitive Impairment Patients Progressing to Alzheimer’s Disease

**DOI:** 10.3390/biomedicines10071713

**Published:** 2022-07-15

**Authors:** Yunxing Ma, Julia Brettschneider, Joanna F. Collingwood

**Affiliations:** 1 School of Engineering, University of Warwick, Coventry CV4 7AL, UK; Y.Ma.17@warwick.ac.uk; 2 Department of Statistics, University of Warwick, Coventry CV4 7AL, UK; Julia.Brettschneider@warwick.ac.uk

**Keywords:** Alzheimer’s disease, cerebrospinal fluid, tau, amyloid beta, mild cognitive impairment, biomarker, systematic review, meta-analysis

## Abstract

Reported levels of amyloid-beta and tau in human cerebrospinal fluid (CSF) were evaluated to discover if these biochemical markers can predict the transition from Mild Cognitive Impairment (MCI) to Alzheimer’s disease (AD). A systematic review of the literature in PubMed and Web of Science (April 2021) was performed by a single researcher to identify studies reporting immunologically-based (xMAP or ELISA) measures of CSF analytes Aβ(1-42) and/or P-tau and/or T-tau in clinical studies with at least two timepoints and a statement of diagnostic criteria. Of 1137 screened publications, 22 met the inclusion criteria for CSF Aβ(1-42) measures, 20 studies included T-tau, and 17 included P-tau. Six meta-analyses were conducted to compare the analytes for healthy controls (HC) versus progressive MCI (MCI_AD) and for non-progressive MCI (Stable_MCI) versus MCI_AD; effect sizes were determined using random effects models. The heterogeneity of effect sizes across studies was confirmed with very high significance (*p* < 0.0001) for all meta-analyses except HC versus MCI_AD T-tau (*p* < 0.05) and P-tau (non-significant). Standard mean difference (SMD) was highly significant (*p* < 0.0001) for all comparisons (Stable_MCI versus MCI_AD: SMD [95%-CI] Aβ(1-42) = 1.19 [0.96,1.42]; T-tau = −1.03 [−1.24,−0.82]; P-tau = −1.03 [−1.47,−0.59]; HC versus MCI_AD: SMD Aβ(1-42) = 1.73 [1.39,2.07]; T-tau = −1.13 [−1.33,−0.93]; P-tau = −1.10 [−1.23,−0.96]). The follow-up interval in longitudinal evaluations was a critical factor in clinical study design, and the Aβ(1–42)/P-tau ratio most robustly differentiated progressive from non-progressive MCI. The value of amyloid-beta and tau as markers of patient outcome are supported by these findings.

## 1. Introduction

### 1.1. Alzheimer’s Disease in Context

As our global societies evolve, it is well-documented that the average age of the human population is increasing both locally and globally [1]. Age is a significant risk factor for cognitive impairment and dementia [2,3], so increased incidence of these conditions is being seen in association with an ageing population.

Alzheimer’s Disease (AD) is the most common cause of dementia. Clinically observable characteristics of this disorder include memory loss, decline in cognitive function, and changes in behavioral patterns. Further, AD is identified as the greatest cause of death without an effective disease-modifying therapy [4,5]. Efforts to develop drugs to treat AD have had a high failure rate [6].

According to the analysis of the 2000 census and subsequent population projections [7], there were 4.5 million AD patients in the United States (US) in 2000, where 1.8 million of them were ≥85 years old. Hebert et al. estimated that by 2040 there would be 11.0–12.8 million patients (by middle-series or high-series estimation respectively) with over 50% of these being ≥85 years old. Subsequently, Herbert et al. revised these figures in light of the 2010 census [8], refining the estimate to be 11.6 million AD patients in the US by 2040 (where 42.2% of those will be ≥85 years or older), if no preventive measures were developed, and predicting that of the entire population aged ≥85 years old in 2050, 36.6% of them will be affected by AD, and of the population aged 65–74, 3.3% will be affected [8].

To consider Alzheimer’s, it is necessary to define and distinguish the terms Alzheimer’s Disease (AD) and Alzheimer’s dementia. Here we follow the distinction made by Ritchie et al. [9], where they noted that AD describes the underlying pathology throughout the disease course, whereas Alzheimer’s dementia is used to describe the final stages of AD. When attempting to distinguish normal aging from very early-stage Alzheimer’s Disease, and in turn Alzheimer’s Disease, the continuous progression of clinical symptoms and the manual selection of threshold values to distinguish each disease stage, can be difficult and subjective [10]. Of the sporadic and familial AD patient populations, only 5% of these patients exhibit known forms of familial AD [11].

### 1.2. Early-Stage Diagnosis of AD

It might be argued that the failure to date to develop an effective treatment for AD is in part because by the time a patient is diagnosed with AD they are too advanced towards the later stages of the disease. So, the importance of developing a strategy to detect or predict which patients with mild cognitive impairment (MCI, as defined in Section 2.4), or even those who appear cognitively normal, are likely to progress to AD, is receiving increasing attention. To achieve clinical diagnosis of the conversion from MCI to AD remains very challenging. The identification of a truly AD-dominated hallmark is critical in this area [12].

### 1.3. Current Status of AD Diagnosis versus AD Prediction

Presently, a tremendous range of methods exist to differentiate AD patients from healthy controls, comprising both physiological measurements (including imaging) and clinical screening tests. On the other hand, many of these methods lack reliability or robustness; few of them have been well-validated for their target population [13]. To better understand and identify AD, the timeline involved in progression from MCI to two key outcomes (either confirmed AD or a stable non-progressing form of MCI) needs to be better described. Ideally it would be possible to achieve early-stage detection as the foundation and necessary indication for early intervention, to delay, relieve, or even prevent AD in those who are pre-symptomatic.

Different cognitive screening tests have been shown to have different reliability coefficients (both internal and inter-rater consistency). Additionally, not all assessments include a quantified performance indicator [14]. The essential features that any potential techniques should have include reliability and reproducibility [15], and are ideally non-invasive, easy to measure, and low cost. The use of fluid biomarkers continues to hold promise to support aspects of diagnosis, including potential scope to support preclinical detection of AD in cognitively normal older individuals.

### 1.4. Usage of Biomarkers in Prediction and Diagnosis of AD

There is no consensus on the choice of individual or sets of biomarkers used for CSF AD analysis or of their corresponding threshold values [12]. In order to achieve AD prediction in future, three major types of fluid biomarker currently under consideration are directly linked with pathogenic changes associated with AD, specifically: 1. Neurodegeneration associated with total tau (T-tau); 2. The metabolism of amyloid precursor protein (APP) with particular emphasis on the amyloid beta Aβ(1-42) peptide; and 3. The pathology specifically associated with phosphorylated tau (P-tau) intraneuronal tangles [16]. The various models of the AD pathological cascade, incorporating tauopathies and amyloidopathies, reveal different timelines for the evolution of amyloid beta or tau pathologies, where this has been investigated post-mortem [17], and/or tracked in mutation carriers [17,18]. The evolution of Alzheimer’s disease biomarkers is reviewed in detail elsewhere [19].

An important and fundamental assumption informing the work in the present paper is that AD fluid biomarkers follow a sigmoidal form as a function of time [18]. Among these, the aggregation of Aβ(1-42) (stemming from APP cleavage and subsequent cortical amyloid deposition [16]) is a hallmark of AD, and monomeric Aβ(1-42) is found at lowered concentrations in the peripheral fluids of AD patients [16,20].

It has been suggested that abnormally high values of T-tau are caused by neuronal damage. In AD patients, cortical neuronal loss has been linked with elevated concentrations of T-tau in CSF [21,22,23], and high CSF T-tau concentrations have been clinically correlated with rapid cognitive decline in AD and show utility as a marker of disease stage [24].

Phosphorylated(P)-tau is an abnormal form of tau found in AD and unlike normal tau it is sedimentable. During the development of AD it is understood that P-tau self-assembles into paired helical filaments admixed with straight filaments leading to the formation of neurofibrillary tangles [25]. P-tau levels correlate with cortical tangle formation and reflect the phosphorylation state of tau [21,26]. Elevated concentrations of P-tau181 were previously associated with rapid cognitive decline in AD [24], and more recently the P-tau217 residue has started to be evaluated alongside P-tau181 as an indicator of amyloid and tau pathology in clinical settings [27].

### 1.5. Overview of Study

In the following work, a systematic review and quantitative meta-analyses were performed to test relationships between these three potential biomarkers in CSF (Aβ(1-42), T-tau, and P-tau) and the evolution of AD in longitudinal evaluations of levels relative to baseline, using prior-published experimental data. The primary focus of the analysis was on the period describing the transition of a patient from MCI to AD, where it is critical to discover the main biomarker characteristics that differentiate patient outcomes for those who have a stable form of MCI, and those who progress to a confirmed diagnosis of AD. We report highly statistically significant differences (*p* < 0.0001) for the standard mean difference for all six meta analyses performed in this study, confirm that those MCI patients who were stable tended to have slightly higher Aβ(1-42) levels than healthy controls, and that levels were significantly lower in MCI patients who progressed to develop Alzheimer’s disease. The opposite was observed for P-tau and T-tau levels, where MCI patients progressing to develop Alzheimer’s disease exhibited the highest levels compared to non-progressive MCI and healthy controls. The data suggest that using these markers, the Aβ(1-42)/P-tau ratio gives the most robust indicator of a patient transitioning from MCI to AD, and the follow-up period for longitudinal evaluations is identified as especially critical to clinical study design for this purpose.

## 2. Materials and Methods

### 2.1. Search Strategy

**Figure 1:**
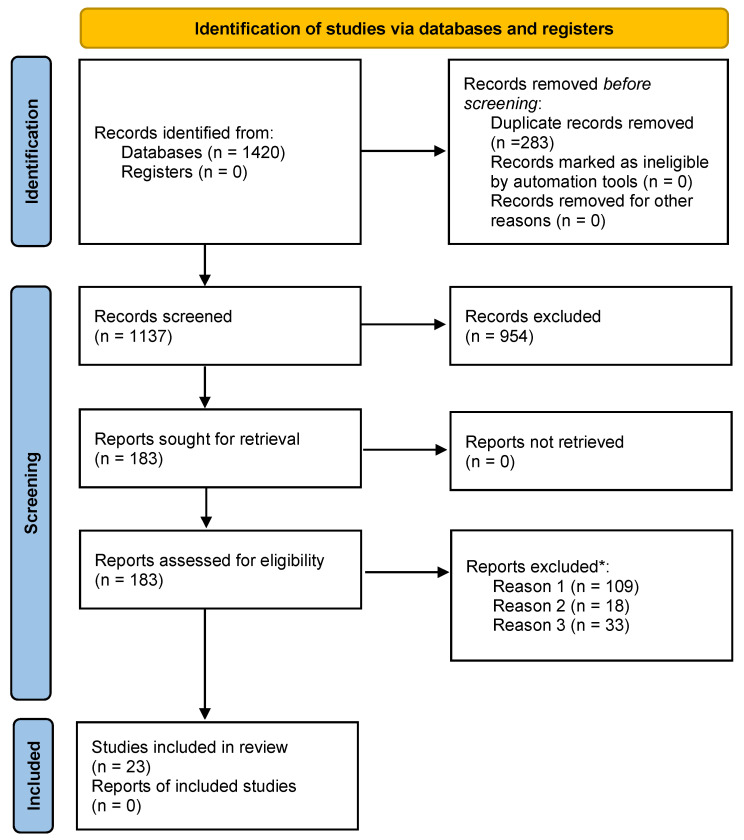
Flow chart of the systematic literature search according to PRISMA 2020 guidelines [28], using the PubMed and Web of Science databases as detailed in the main text, for records published in or after 1994, with the record identification (data extraction) completed in April 2021. * For reports excluded for multiple reasons, only the primary exclusion criterion is counted here (i.e., each excluded report is only counted once). The primary reasons for exclusion of reports were as follows. Reason 1: Diagnostic outcome information was insufficient (did not explicitly consider the transition from Mild Cognitive Impairment to Alzheimer’s disease); Reason 2: Data parameters prevented inclusion (e.g., an incomplete dataset for the purpose of this study, or because data could not be converted into mean ± standard deviation at baseline such as where ratios between markers were reported, or because the population sampled was too small <30); Reason 3: Analysis in a report replicated that in one or more other reports meeting the inclusion criteria (e.g., where there were multiple studies evaluating the ADNI dataset, or the report was a review of other studies).

We followed the Preferred Reporting Items for Systematic Reviews and Meta-Analyses (PRISMA) guidelines. The study is registered in INPLASY; registration number 202270020. The systematic review, and subsequent meta-analyses, were performed as described in Figure 1 and Appendix A respectively, to include papers extracted from the PubMed and Web of Science databases, where these were published in English and dated 1994 onwards (to include works incorporating the association of APP with Alzheimer disease).

The review specifically focuses on longitudinal (instead of cross-sectional) studies that included measurements repeated after baseline at one or more timepoints, so that levels can be tracked in a cohort. The clinical diagnoses reported according to recognized criteria were accepted for the purposes of this analysis, rather than requiring validation in the form of neuropathological assessment at autopsy. Further, this study only focuses on Alzheimer’s Disease, so progression from MCI to disorders other than Alzheimer’s (including Parkinson’s related disorders) is excluded.

The following search query terms were used in PubMed (‘Title’) and Web of Science (‘Topic’) respectively, with the search being completed in April 2021:

((((Alzheimer) OR (AD) OR (MCI) OR (mild cognitive impairment)) AND ((CSF) OR (Cerebrospinal fluid)) AND ((biomarker) OR (iron) OR (metal)) AND ((longitudinal) OR (follow-up))) NOT (Review)) NOT (Parkinson).

### 2.2. Inclusion and Exclusion Criteria

Studies measuring CSF levels of Aβ(1-42) and/or tau (T-tau, and/or P-tau) were included. It was also necessary for the studies to include the values for these analytes at baseline (initial visit) and then at one or more subsequent time points for the healthy controls and the patients along with their diagnostic status (MCI, AD, or another form of dementia). To be included, the studies also needed to state which criteria were used for the diagnosis of MCI and AD. For CSF analyte levels, the study needed to document not only the average values but also the standard deviation or interquartile range for each measurement. The study population also needed to be stated for each diagnostic group. Studies that did not meet these criteria were excluded: for example, Zetterberg et al. [29] was excluded as analyte concentration data were reported with 95% confidence interval and, therefore, did not include the necessary statistical information to be included in the present analysis.

Where multiple independent studies were performed with a cohort, all studies except for the latest available study were excluded on the basis that the latest study should contain the most up-to-date information and methods. (Notably, for the ADNI dataset, papers except for Spencer et al.’s 2019 study [30] were excluded. For example, Cui et al. [31] was excluded because this study used the same dataset from ADNI as Spencer et al. subsequently did in 2019). The methods used to assess cognitive function (e.g., MMSE) were not taken into account in the inclusion/exclusion criteria, but the analytical methods for the measurement of the analytes were examined, and for the example of the Malmö University Hospital’s dataset, both xMAP and ELISA methods were used; after evaluation it was determined that both should be retained in our analysis [32,33].

Studies of MCI patients progressing to forms of dementia that were not explicitly classified as AD were excluded. Studies that did not include measures of CSF T-tau, P-Tau, or Aβ(1-42) were also excluded. Additionally, studies were excluded where the data were reportedly providing the confidence interval of the median, which prevented inclusion because those data could not then be transformed into the mean and standard deviation.

After application of the exclusion and inclusion criteria, 23 studies in total were included. Of these, 22 provided measures of amyloid in CSF, 20 provided measures of T-tau, and 17 provided measures of P-tau. Of the 17 studies providing P-tau measures, 13 explicitly stated that this was P-tau181, and in the remaining four studies the measurement of P-tau181 was indicated or implicit from the date of the report [34], choice of assay [35], or the literature cited in connection with the P-tau measure [36,37].

### 2.3. Statistical Analysis

The format of the data, as provided in the source papers, sometimes gave direct access to the average and standard deviation values for the analytes, but in other cases it was in the form of the median and accompanying range, or median with first and third quartile values. In the latter cases, the method previously published by D. Luo and X. Wan [38,39] was used to estimate the average and standard deviation to align the datasets to enable a meta-analysis. Where the source data were available as the median and inter-quartile range, they were assumed to follow a normal distribution with standard deviation equal to the inter-quartile range divided by 1.35.

Hedges’ g values were calculated to determine the effect size for the datasets included in this study, using a random effect size model. In this context, the Hedges’ g value was defined as positive for the scenario where patients with progressive MCI had a higher CSF baseline measurement than the other groups with which they were compared (non-progressive MCI or health control) for each selected biomarker. To detect publication or other biases, funnel plots were used. Heterogeneity across studies was primarily accessed by Higgins’ I^2^.

Full details of the meta-analysis are set out in Appendix A.

### 2.4. Use of Diagnostic Criteria to Define MCI and AD

**Figure 2:**
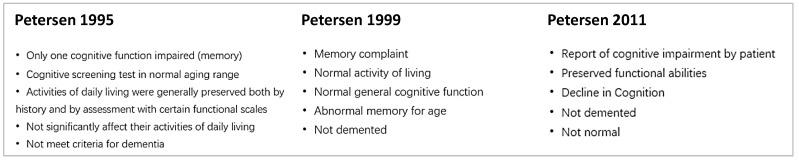
Evolution of diagnostic criteria for MCI from 1995 to 2011 [40,41,42].

The majority of the studies included in the systematic review exclusively used Petersen’s criteria for MCI for diagnostic purposes. For the period of this review (since 1994), we note the evolution of Petersen’s criteria from 1995 to 2011 as summarized in Figure 2 [40,41,42].

In the evolution of the diagnostic criteria for MCI, it was mentioned in Petersen’s 2011 criteria that MCI could be non-amnestic or amnestic [42]. Under the early criteria for diagnosing MCI, the term “MCI” was equivalent to “amnestic MCI” in the latter definition. Overall, however, it appears that the diagnostic criteria have been generally consistent and are not expected to introduce a significant systematic error in the context of the present analysis. Herukka and co-workers used the CDR (clinical dementia rating) score, where patients were diagnosed as MCI if they had a CDR score of 0.5 and were performing below the age-adjusted norms in at least one cognitive domain in any of memory, language, attention, and executive function or global function [34]. Brys and co-workers used CDR score and GDS (Global Deterioration Scale) for the diagnosis of MCI [43].

In determining the diagnostic criteria for AD, all the research included in this study used at least one of the following criteria: NINCDS-ADRDA, DSM-IV, or DSM-III-R. Hansson and Buchhave combined DSM-III-R with NINCDA-ADRDA as Buchhave’s work was an extension of follow-up of Hansson’s [32,44].

## 3. Results

### 3.1. Methods to Determine Analyte Concentration in CSF

There are two major types of CSF analysis method reported in the studies identified in the systematic review. These are xMAP (multi-analyte profiling) and ELISA (enzyme linked immunosorbent assays) method. In presenting the results graphically, the findings were sorted first by analysis method (xMAP or ELISA) and then by year of publication. Both methods provide reported values as an absolute value (the mass of analyte per unit volume of CSF), and as longitudinal change is the parameter of interest, both xMAP and ELISA data are included here as reported in the source papers.

### 3.2. Baseline CSF Biomarker Measurements

**Figure 3:**
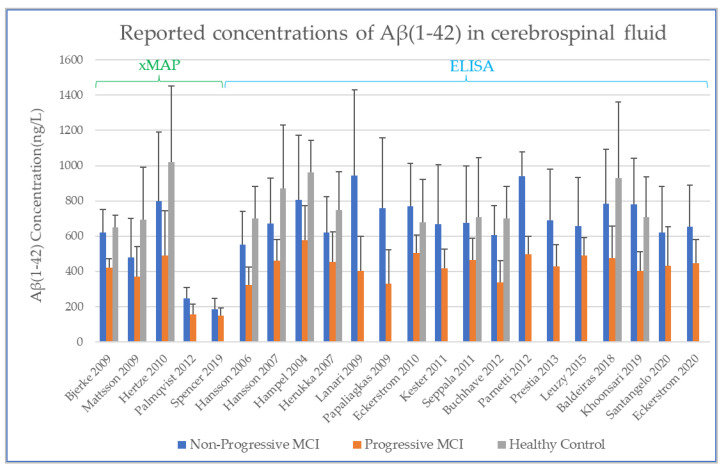
Concentration of amyloid beta 1-42 in CSF at baseline, as determined by xMAP and ELISA.

**Figure 4:**
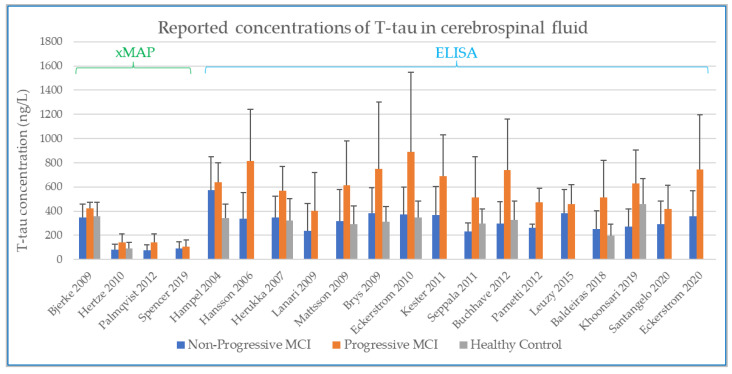
Concentration of T-tau in CSF, as determined by as determined by xMAP and ELISA.

**Figure 5:**
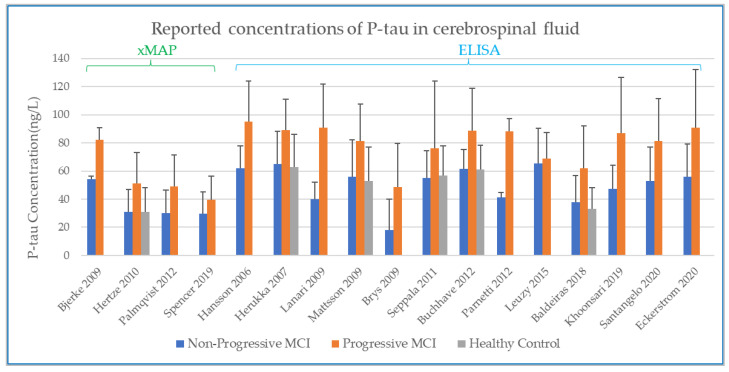
Concentration of P-tau in CSF at baseline, as determined by xMAP and ELISA.

The values at baseline from the studies identified in the systematic review are shown below for Aβ1-42 (Figure 3), T-tau (Figure 4), and P-tau (Figure 5). The error bars in each plot show the standard deviation, and only the upper bound of the standard deviation (>mean) is shown [30,32,33,34,35,36,37,43,44,45,46,47,48,49,50,51,52,53,54,55,56,57,58].

In comparing the studies performed by xMAP and ELISA, there is, by observation, evidence of systematic differences between the concentrations detected with the two different analytical methods. The advantage of using xMAP for CSF analyte measurement is that it enables simultaneous tracking of multiple biomarkers. Compared to the classical single-biomarker ELISA technique, xMAP reduces working time and still achieves correlations for P-tau and T-tau but not for Aβ42 [12,59]. However, it does present a problem for the comparison of absolute values between studies. In examining published data, we found moderate correlations between ELISA and xMAP measurements [59] of Aβ1-42 (r = 0.47) and stronger correlations for tau (r = 0.87 for P-tau, r = 0.96 for T-tau). Shaw and co-workers reported that concentrations measured by ELISA equate to ~200% of Aβ1-42, ~400% of T-tau, and ~125% of P-tau as measured by xMAP [60]. The same trend is observed in the data we present in Figure 3, Figure 4 and Figure 5, where the greatest difference in reported concentrations for xMAP versus ELISA measurements is evident for T-tau (Figure 4). We compared the xMAP analytical approach in these T-tau measures. Spencer and co-workers [30] used data from ADNI, where the CSF sample analysis is described according to the published ADNI protocol [30]. For Hertze’s analysis of baseline CSF, the patients were from Malmo University Hospital [47]. The systematic difference between xMAP technology and ELISA measurement was adjusted for by some (e.g., Bjerke, Mattsson, Hertze, Figure 3) but not necessarily by others (e.g., Palmqvist, Spencer, Figure 3), leading to a big offset in the values reported by xMAP for Aβ1-42. Indeed, in the study by Palmqvist, the effect of xMAP on absolute concentrations is not discussed [48]. While there is no accurate conversion factor to equate xMAP and ELISA data, the important factor in this present study is the relative change in each analyte within a group, so here it is deemed valid to retain both the xMAP and ELISA data for the analyte comparisons between healthy controls (HC), non-progressive MCI (Stable_MCI), and MCI that progresses to AD (MCI_AD).

### 3.3. Aβ1-42/T-Tau and Aβ1-42/P-Tau Ratio

**Figure 6:**
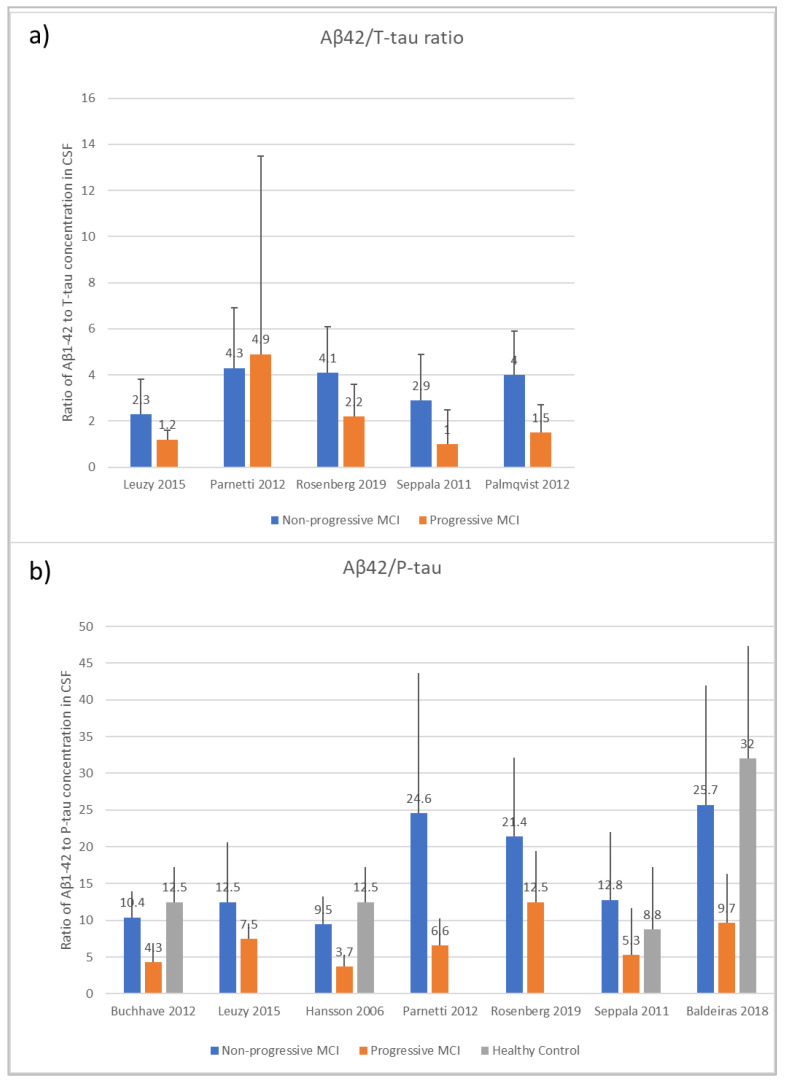
The ratio of Aβ1-42 to (**a**) T-tau and (**b**) P-tau in CSF, using data for mean and standard deviation (upper bound shown as error bar) taken directly from the sub-set of papers in the systematic review that included this information [32,37,44,48,54,55,57,61].

As shown in Figure 6, a number of studies report ratios of Aβ42/T-tau and Aβ42/P-tau. This data format does not enable the use of the data in the following meta-analyses, except where there was access to the separate datasets for the three analytes, so the ratio information shown here is retained for discussion purposes but not included in subsequent calculations.

### 3.4. Follow-Up Duration

There are marked differences in study duration for the different studies included in this work. The length of follow-up was converted into the format mean ± standard deviation using the method described in Section 2.3. In several papers, only the maximum length of follow up duration was provided, which prohibited follow-up duration being converted into the required format.

There is no standard follow-up duration for such studies, and this constrains analysis and interpretation. For example, it is unclear whether the patients are studied over a sufficiently long period that they might progress to fully develop the syndrome of AD. Some reports in the field do not even record the follow-up duration. The effect of follow-up period on the prediction accuracy and threshold value is considered in detail in the Discussion.

### 3.5. Meta Analysis of Studies Investigating the Association between Levels of Amyloid Beta and Tau in CSF, and Progression to Alzheimer’s Disease

We conducted meta-analyses comparing non-progressive MCI (stable MCI) with progressive MCI (MCI_AD) for each of the three analytes (amyloid beta 1-42, ‘amyloid’; T-tau, and P-tau), and did likewise to compare healthy controls (HC) with MCI_AD. The key results from these six separate meta-analyses are summarized in Table 1.

For each of the comparisons, a separate meta-analysis was conducted for the three different analytes, and these are described in full in Appendix A. Effect sizes were determined using random effects models, ensuring somewhat balanced weights across studies despite the inclusion of individual studies with much larger sample sizes than all others and considering differences in the populations of the individual studies. Roughly symmetric funnel plots confirmed that there is no clear evidence of bias in any of the comparisons. The heterogeneity of effect sizes across studies was confirmed with very high significance for all three meta-analyses comparing Stable_MCI versus MCI_AD (*p* < 0.0001), as well as for amyloid in the HC versus MCI_AD comparison (*p* < 0.0001), and a statistically significant difference was also observed for T-tau for HC versus MCI_AD (*p* < 0.05), although not for P-tau. Hence, as justified in Appendix A, random effects are used for all but the last comparison. Note that the small number of studies in the last condition means that we can only draw limited conclusions from this.

Effect sizes are given in standard units. In most of the comparisons, the absolute magnitude of the effect is between 1 and 1.2 standard error difference, except for amyloid in HC vs. MCI_AD, where it is even higher with 1.73. The direction of the effects also confirms the trends reported earlier in Figure 3, Figure 4, Figure 5 and Figure 6: amyloid is increased in both non-AD conditions (Stable_MCI and HC) versus MCI_AD, while the other two analytes (T-tau, P-tau) are decreased.

Normality assumptions were checked in each of the six settings and turned out to be sufficiently satisfied (supported by the histogram plots showing the distribution of effect sizes for each analysis in Appendix A Figure A1, Figure A3, Figure A5, Figure A7, Figure A9, Figure A11), while the symmetry of the funnel plots created for each of the conditions (Appendix A, Figure A2, Figure A4, Figure A6, Figure A8, Figure A10, Figure A12) confirmed no clear evidence for bias.

**Figure 7:**
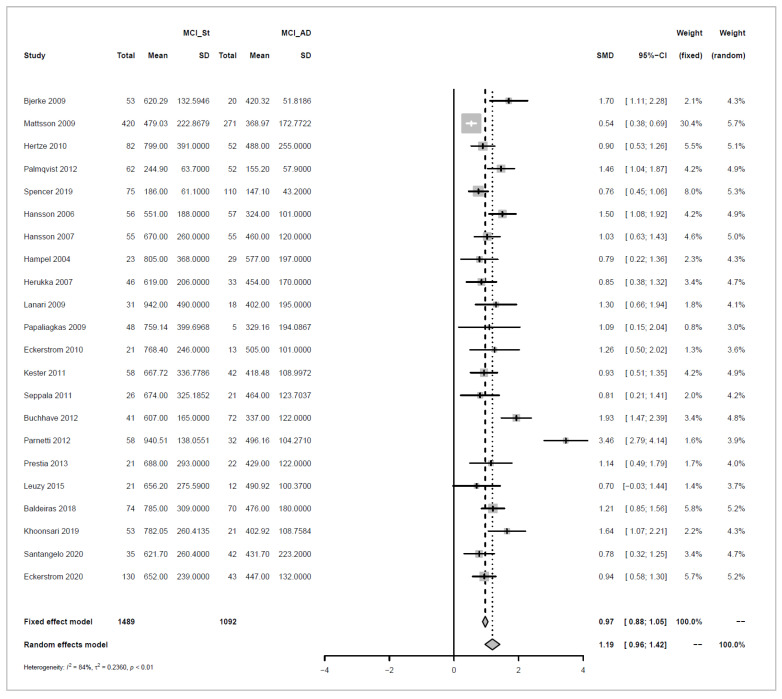
Meta-analysis of studies investigating CSF Aβ1-42 levels for non-progressive MCI (MCI_St) versus progressive MCI (MCI_AD), see Appendix A.2.1 [30,32,33,34,35,36,37,44,45,46,47,48,49,50,51,52,53,54,55,56,57,58].

**Figure 8:**
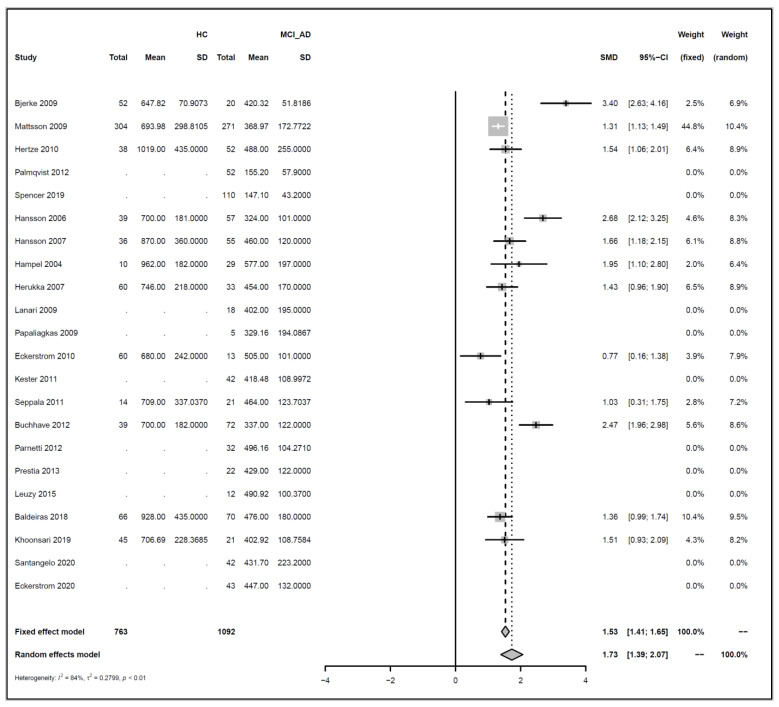
Meta analysis of studies investigating CSF Aβ1-42 levels for healthy control (HC) versus progressive MCI (MCI_AD), see Appendix A.2.2 [30,32,33,34,35,36,37,44,45,46,47,48,49,50,51,52,53,54,55,56,57,58].

**Figure 9:**
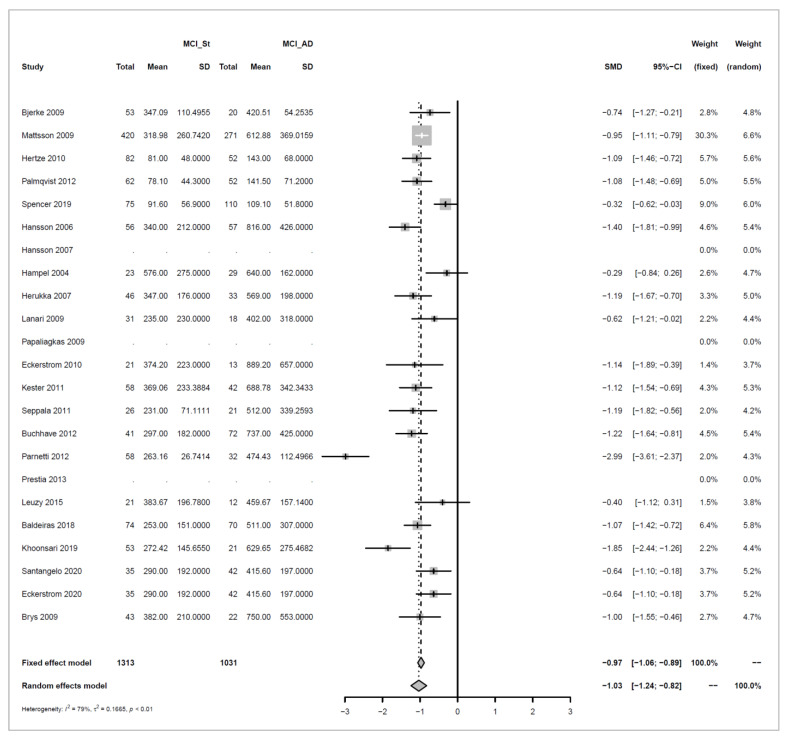
Meta-analysis of studies investigating CSF T-tau levels for non-progressive MCI (MCI_St) versus progressive MCI (MCI_AD), see Appendix A.3.1 [30,32,34,35,36,37,44,45,46,47,48,49,50,52,53,54,55,57,58].

**Figure 10:**
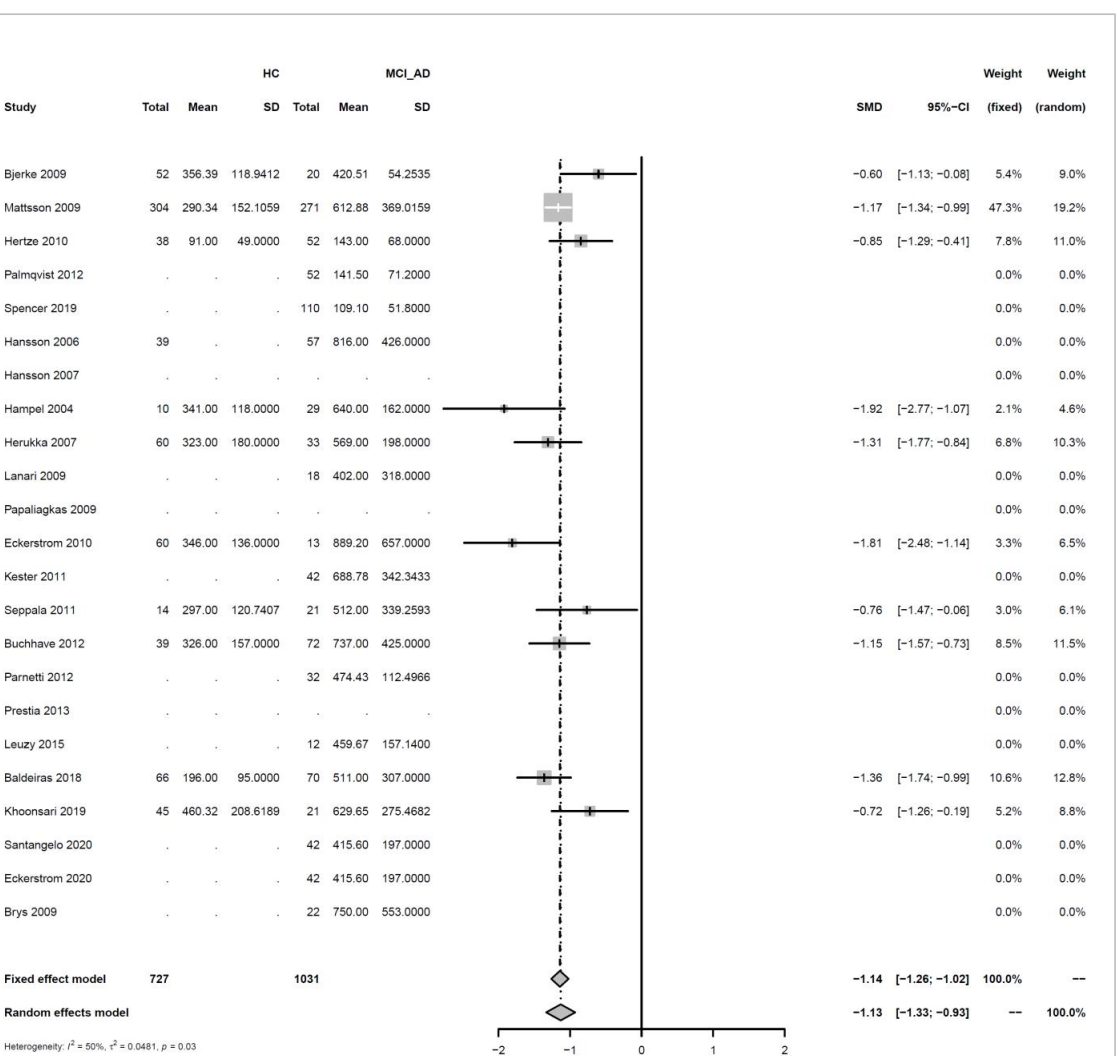
Meta analysis of studies investigating CSF T-tau levels for healthy control (HC) versus progressive MCI (MCI_AD), see Appendix A.3.2 [30,32,34,35,36,37,44,45,46,47,48,49,50,52,53,54,55,57,58].

**Figure 11:**
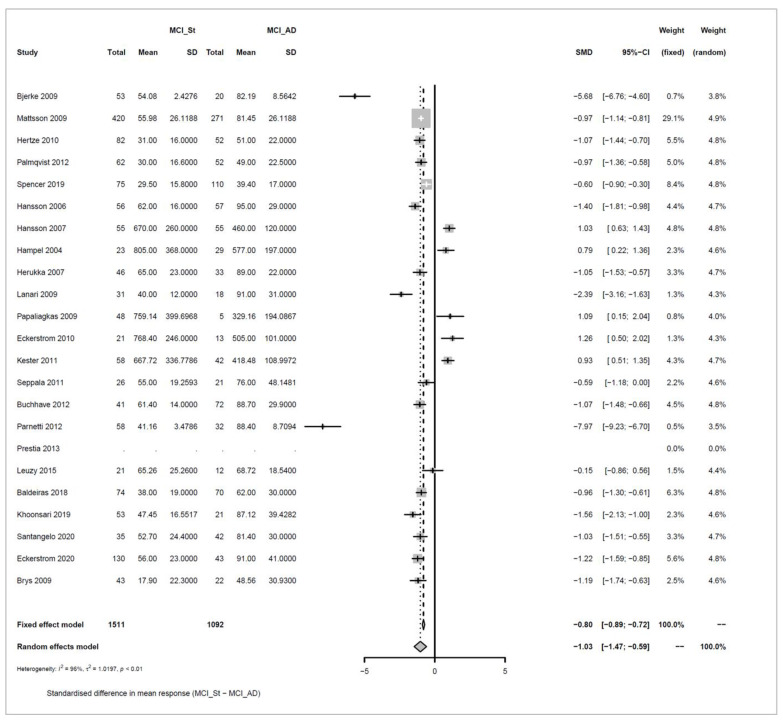
Meta-analysis of studies investigating CSF P-Tau levels for non-progressive MCI (MCI_St) versus progressive MCI (MCI_AD), see Appendix A.4.1 [30,32,34,35,36,37,44,45,46,47,48,50,54,55,57,58].

**Figure 12:**
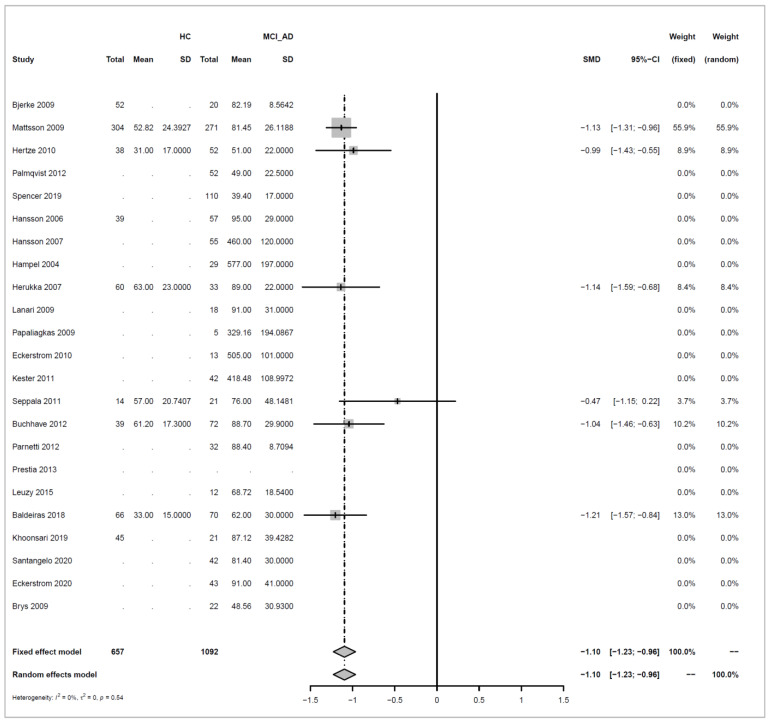
Meta analysis of studies investigating CSF P-tau levels for healthy control (HC) versus progressive MCI (MCI_AD), see Appendix A.4.2 [30,32,34,35,36,37,44,45,46,47,48,50,54,55,57,58].

Forest plots illustrating the findings for each meta-analysis are shown below as Figure 7, Figure 8, Figure 9, Figure 10, Figure 11 and Figure 12.

Concerning the values of the three analytes with respect to baseline values, we observed that CSF Aβ and tau levels were differentiated by patient outcome. With either xMAP or ELISA, MCI patients who did not progress to AD (non-progressive MCI) tended to have slightly higher Aβ(1-42) levels than healthy controls (*p*-value < 0.001). Levels were significantly lower in MCI patients who progressed to AD (progressive MCI). The opposite, consistent with prior observations, was observed for P-tau and T-tau where progressive MCI patients exhibited the highest levels compared to non-progressive MCI and healthy controls.

## 4. Discussion

### 4.1. Limitations of Current Study

A constraint of the data available is that the raw data did not enable analyte trajectories to be plotted as a function of time for individual patients. The individual studies that were used as the source papers for this analysis constrained experimental designs that justify our decision to undertake the separate meta-analyses comparing two conditions in each. In principle, we might alternatively have performed a meta-analysis with three conditions (Stable_MCI, MCI_AD, and HC) using analysis frameworks developed for multiple treatment designs. In practice, this would have significantly reduced the number of studies that could be incorporated into the meta-analysis, and the priority here was to make the fullest possible use of the available data. On this occasion, the *p*-values are self-evidently so small that the statistically significant differences between the groups would be preserved after any standard adjustment for multiple testing (Bonferroni).

Historically it has been observed that CSF Aβ1-42 concentrations are lower in AD patients than in healthy controls and MCI patients. In the papers systematically reviewed for this study, it is noted that all progressive MCI (MCI_AD) patient cohorts have lower average baseline Aβ1-42 concentration than the non-progressive (stable MCI) patients. However, a limitation was that there was no way of predicting clinically, at the time of the baseline measurement, whether MCI patients would progress to develop AD. This points to a fundamental drawback, which is to determine whether a specific patient meets the criteria for AD is entirely based on clinical diagnosis. This creates scope for circular reasoning, where the accuracy of early prediction or diagnosis based on CSF biomarkers is totally dependent on the clinical diagnostic criteria used.

A further limitation of this analysis is that the participants in the systematically-reviewed studies were selected for those studies, and it remains to be tested whether these participants were truly representative of wider (global) populations. Access to CSF samples is limited to those who were referred to take part in these clinical studies, typically because of concern about cognitive impairment, so access to the referral process introduces bias.

Lastly, over the period evaluated (since 1994), P-tau measures defaulted to being of P-tau181. By comparison, P-tau217 is a novel candidate biomarker, so it could not be incorporated into this analysis. Of the included studies reporting P-tau measures, 13 explicitly evaluated P-tau181, and it is an assumption (as detailed in Section 2.2) that for the four studies where this was not explicitly stated only P-tau181 residues could have been evaluated. Both P-tau181 and P-tau217 are currently being shown to have strong potential to differentiate patients with AD from other neurodegenerative disorders [62] and the relationship between P-tau181, P-tau217, and amyloid burden is also being examined [27]. In many respects, changes in P-tau217 levels appear to parallel P-tau181 changes, and P-tau181 remains a valuable marker [27]; it will, however, be interesting to see how P-tau217 performs (including the Aβ1-42/P-tau217 ratio) in future meta-analyses for the purpose of predicting progression from MCI to AD.

### 4.2. The Effect of Follow-Up Length

Within some of the papers included in this systematic review, healthy control (HC) groups are omitted. Our observation from the results in this review is that the CSF Aβ1-42 concentration for non-progressive MCI (Stable MCI) is more similar to HC, with higher values than reported for progressive MCI (MCI_AD).

**Figure 13:**
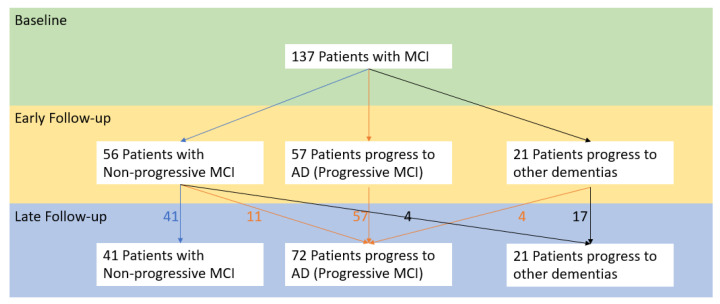
Illustrating the diagnostic trajectories for the 137 patients included in the studies summarized in Table 2 (derived from Hansson et al. [32] and Buchhave et al. [44]).

In Table 2, we show an analysis of data concerning the values of the three CSF analytes for patients in a longitudinal study initiated by Hansson and extended by Buchhave et al. [32,44]. From the data summarized in Table 2a,b, we extrapolated the values (Table 2c) for those patients who had been identified as stable MCI at early follow up, but who transpired to have progressed to Alzheimer’s disease or another form of dementia at a later follow-up. The diagnostic trajectories for these cohorts are illustrated in Figure 13.

The key point here is that in Hansson’s early follow-up [32], 15 patients who were previously classified as MCI according to Petersen’s criteria had been classified as having dementia in Buchhave’s late follow-up [41,44]. They are henceforth referred to as late-progressive-MCI patients here. Of these 15 diagnosed with dementia, 11 were confirmed with AD. From our analysis included in Table 2, it can be observed that the baseline CSF Aβ1-42 concentrations for these 15 patients were closer to the values for progressive MCI (MCI_AD) at the early follow-up, and for P-tau they were closer to non-progressive MCI (stable MCI).

In context with the systematic review results from baseline CSF measurements in this study, our analysis of the longitudinal studies by Hansson and Buchhave support the conclusion that lower Aβ1-42 concentrations in individuals with an MCI diagnosis are more likely to develop AD. A longer follow-up period would increase the chance of identifying those MCI patients at risk of developing AD; we hypothesize that a longer follow-up interval would be associated with greater separation in the Aβ1-42 concentrations for non-progressive versus progressive MCI patients.

We note from the analysis presented in Table 2 that at baseline, the average P-tau concentration in these 15 late-progressive MCI patients is closer to that for non-progressive MCI patients. For T-tau, the group average concentration at baseline in these 15 patients is approximately mid-way between the values for the non-progressive and progressive subgroups. It will be interesting to see if this finding is reproduced in larger cohorts in future studies.

**Figure 14:**
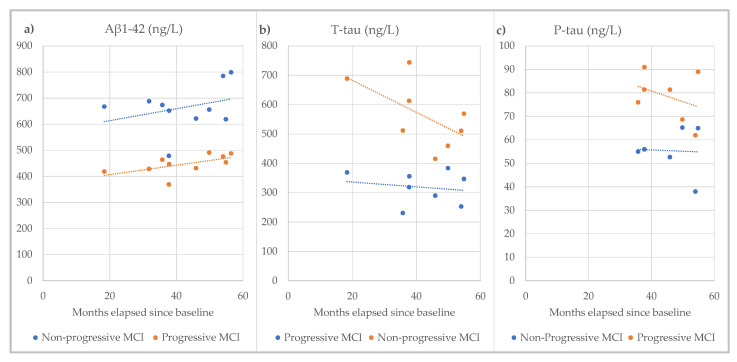
Showing the relationship between analyte value and months elapsed since baseline for (**a**) Amyloid, (**b**) T-tau, and (**c**) P-tau from the subset of studies in this systematic review that included follow-up duration [34,36,37,46,47,53,54,56,57,58].

For the studies in this systematic review that quantified the follow-up interval for non-progressive MCI patients, the values for the three analytes are summarized in Figure 14. Each study included here followed a similar protocol with a baseline measurement and regular assessment of cognitive status, usually at 6 monthly intervals. Importantly, the trends in these results from the overall cohort are consistent with the conclusions drawn from the longitudinal study detailed in Table 2 and Figure 13.

In conclusion, all six meta-analyses showed statistically significant SMD (*p* < 0.0001) and reported values for the Aβ(1-42)/P-tau ratio indicated this as the most robust indicator of a patient transitioning from MCI to AD. The follow-up period for longitudinal evaluations was identified as especially critical to clinical study design, and based on the evidence in this analysis, extending this follow-up period should lead to greater separation in the analyte values for progressive versus non-progressive MCI patients. While the roles of amyloid-beta and tau continue to be debated, their value as markers of patient outcome is supported by these findings.

## Figures and Tables

**Table 1:** Summary of key findings from the six meta-analyses described in Appendix A**.**

		Number of Individual Studies	Effect Size (Random Where Applicable)		Measures of Heterogeneity		Test for Heterogeneity	
			SMD	*p*	tau^2^	I^2^	Q	*p*
**Stable_MCI vs. MCI_AD**	**Amyloid**	22	1.19	<0.0001	0.236	83.8%	129.49	<0.0001
	**T-tau**	20	−1.03	<0.0001	0.167	79.0%	90.45	<0.0001
	**P-tau**	22	−1.03	<0.0001	1.020	95.6%	477.66	<0.0001
**HC vs. MCI_AD**	**Amyloid**	12	1.73	<0.0001	0.280	83.7%	67.35	<0.0001
	**T-tau**	10	−1.13	<0.0001	0.048	50.5%	18.18	0.0331
	**P-tau**	6	−1.10	<0.0001	0	0.0%	4.06	0.5414

**Table 2:** Summary of published findings from (**a**) Hansson et al. [32] and (**b**) Buchhave et al. [44] to determine the analyte values in patients who progress to dementia after early and late follow up, respectively, (**c**) our calculated mean values of the three CSF analytes for the 15 patients who were initially classified as non-progressive (stable MCI) but who in fact were reported as having progressed at late follow up.

(a) Summary of data at early follow-up [32]. Data are given as mean (standard deviation).			
Category (number)	Aβ1-42 (ng/L)	T-tau (ng/L)	P-tau (ng/L)
Non-progressive MCI (*n* = 56)	551 (188)	340 (212)	62 (16)
Progressive MCI (*n* = 57)	324 (101)	816 (426)	95 (29)
**(b) Summary of data at late follow-up** [44]**. Data are given as mean (standard deviation).**			
Category (number)	Aβ1-42 (ng/L)	T-tau (ng/L)	P-tau (ng/L)
Non-progressive MCI (*n* = 41)	607 (165)	297 (182)	61.4 (14.0)
Progressive MCI (*n* = 72)	337 (122)	737 (425)	88.7 (29.9)
**(c) Summary of patients who develop AD and other forms of dementia after being classified as non-progressive at early follow-up** [29,38]**. Data shown are the mean values.**			
Number of patients	Aβ1-42 (ng/L)	T-tau (ng/L)	P-tau (ng/L)
*n* = 15	397	490	63.6

**Figure A1:**
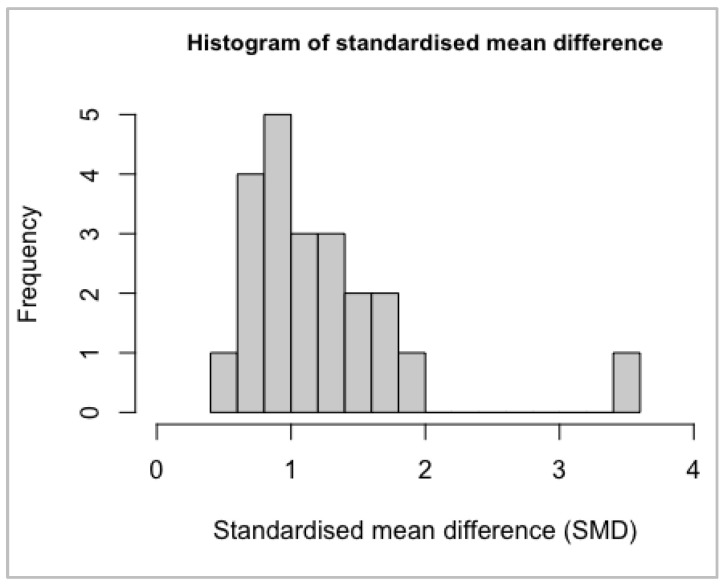
The distribution of the SMD of stable MCI (MCI_St) versus MCI_AD is slightly skewed and shows an outlier, but is otherwise approximately normal.

**Figure A2:**
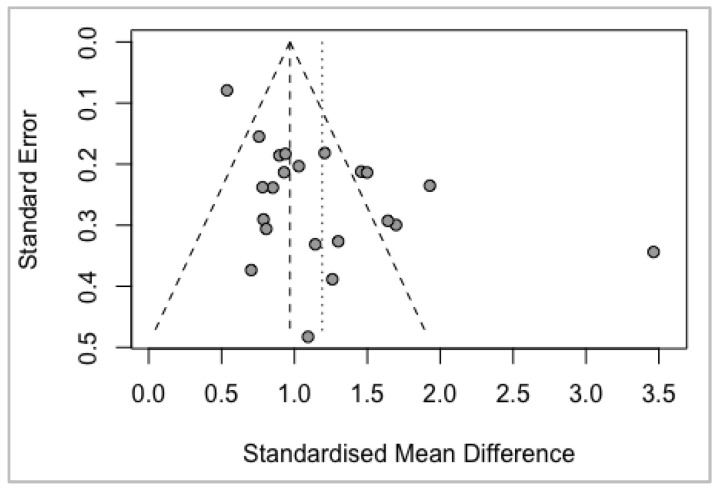
There are a couple of studies outside the confidence bounds for a fixed effect model, including an outlier, but the studies are somewhat balanced on either side of the effect estimates. So, the funnel plot does not reveal an obvious presence of publication or other bias for stable MCI versus MCI_AD.

**Figure A3:**
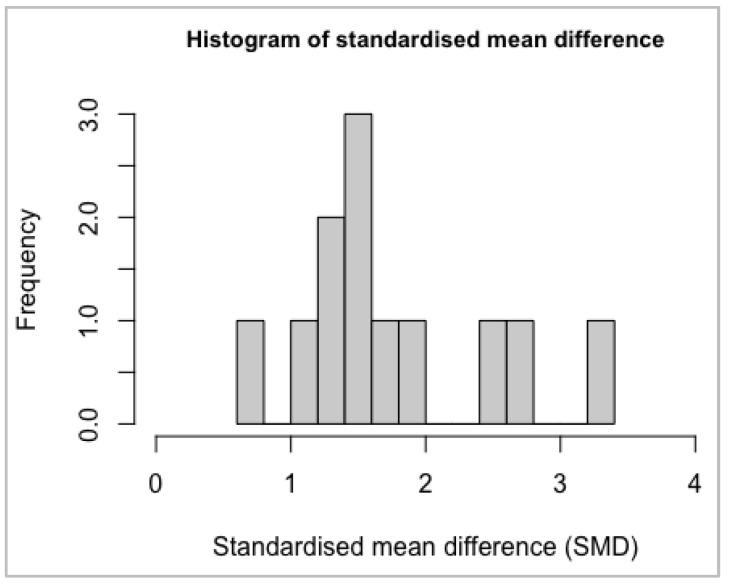
The distribution of the SMD of HC versus MCI_AD is slightly skewed but still approximately normal.

**Figure A4:**
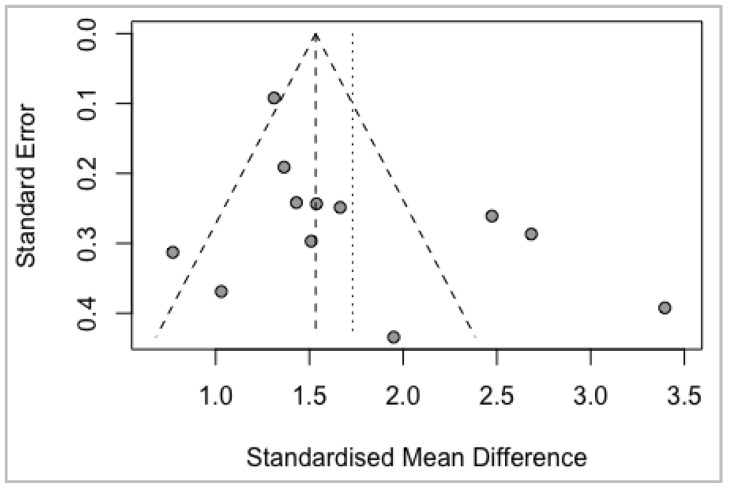
There are a couple of studies outside the confidence bounds for a fixed effect model, including an outlier, but the studies are somewhat balanced on either side of the effect estimates. So, the funnel plot does not reveal an obvious presence of publication or other bias for HC versus MCI_AD.

**Figure A5:**
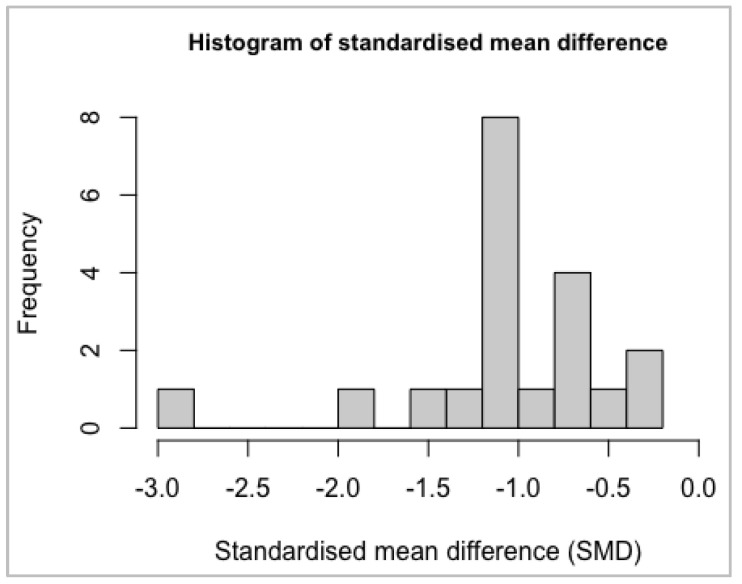
The distribution of the SMD of stable MCI versus MCI_AD is slightly skewed and shows an outlier, but is still within an acceptable range of a normal distribution.

**Figure A6:**
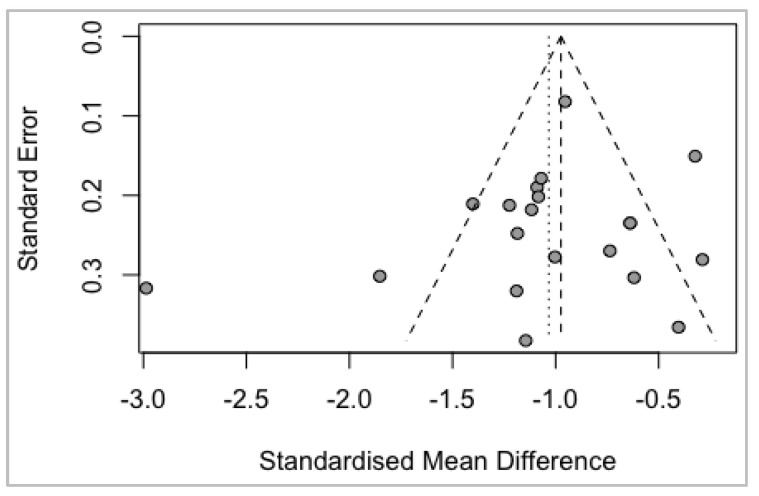
There are a few studies outside the confidence bounds for a fixed effect model including one outlier, but the studies are still well balanced on either side of the effect estimates. So, the funnel plot does not reveal an obvious presence of publication or other bias for stable MCI versus MCI_AD.

**Figure A7:**
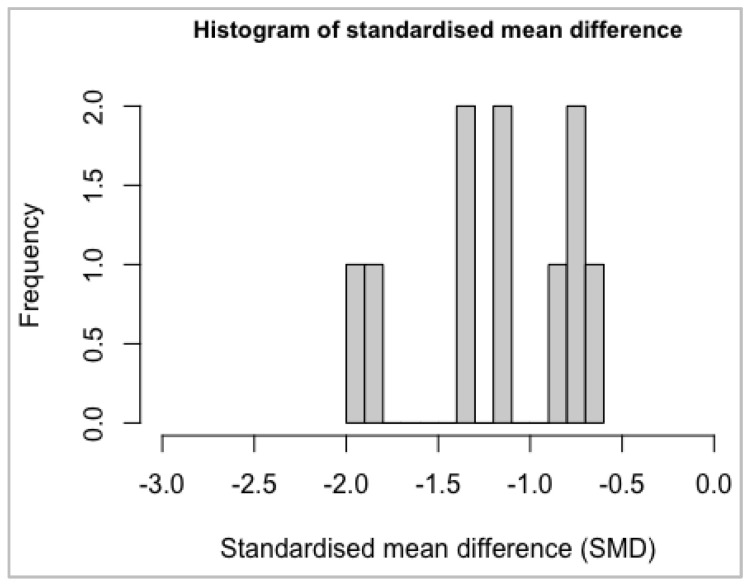
The distribution of the SMD of HC versus MCI_AD is not too dissimilar to a normal distribution, considering the limitations of a small sample.

**Figure A8:**
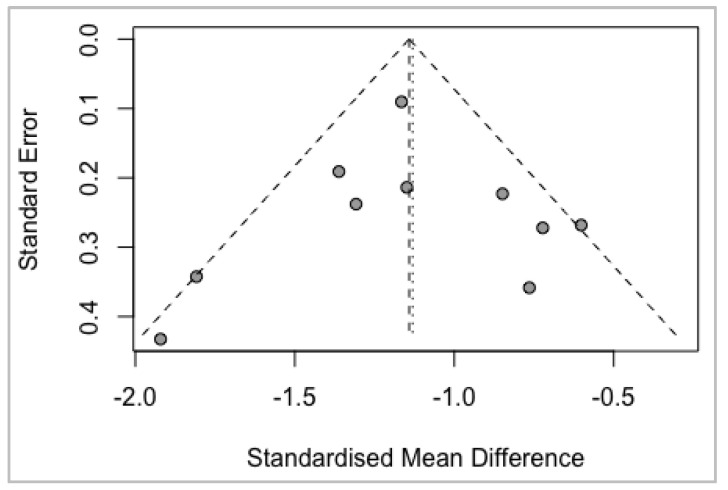
The studies are still well balanced on either side of the effect estimates. So, the funnel plot does not reveal an obvious presence of publication or other bias for HC versus MCI_AD.

**Figure A9:**
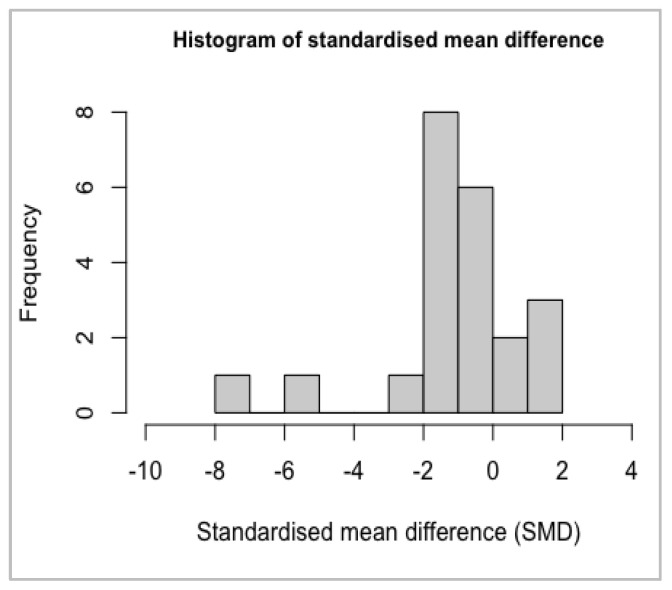
The distribution of the SMD of stable MCI (MCI_St) versus MCI_AD is asymmetric, but not too far from a normal distribution.

**Figure A10:**
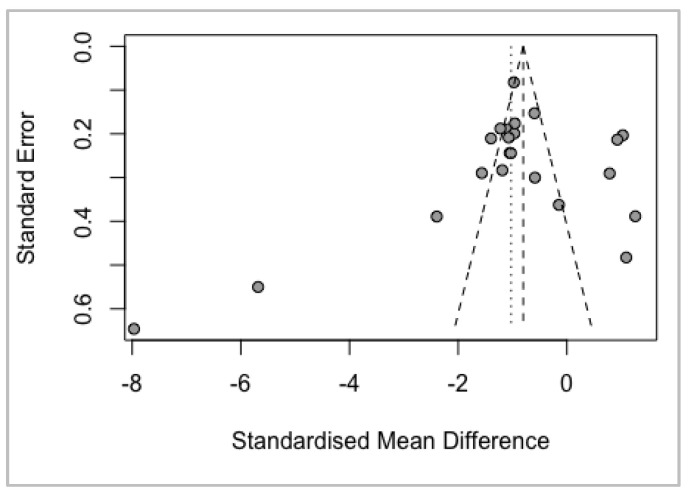
There are a few studies outside the confidence bounds for a fixed effect model, including outliers, but the studies are still somewhat balanced on either side of the effect estimates. So, the funnel plot does not reveal any clear presence of publication or other bias for stable MCI (MCI_St) versus MCI_AD.

**Figure A11:**
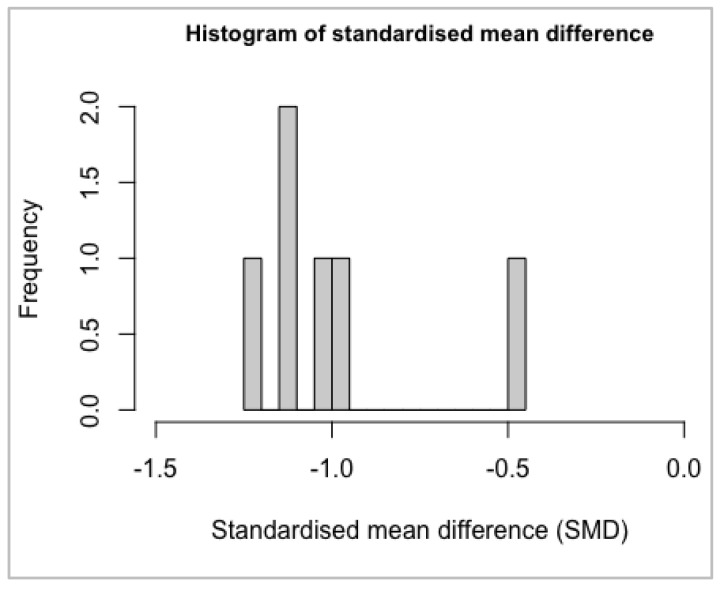
Due to the small number of studies, it is difficult to draw conclusions from the histogram, but there is no clear evidence against the distribution of the SMD of HC versus MCI_AD being approximately normal.

**Figure A12:**
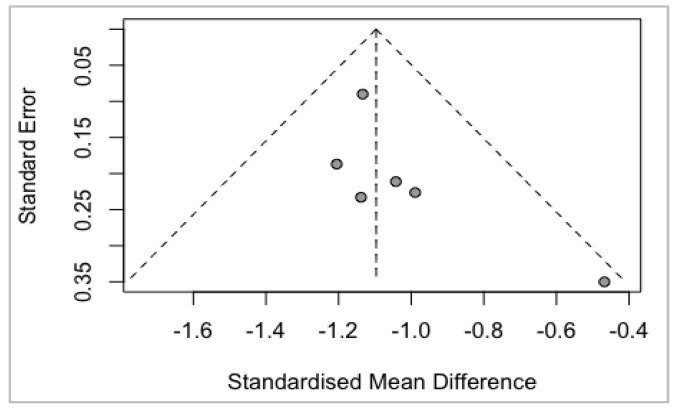
The funnel plot does not reveal any clear presence of publication or other bias, but the number of studies is notably quite small for HC versus MCI_AD.

## Data Availability

The data supporting the reported results are provided as supporting information and can be accessed in their original form in the published studies included in the review and meta-analyses.

## Supplementary Materials

The following is available online at https://www.mdpi.com/article/10.3390/biomedicines10071713/s1, the accompanying PRISMA checklist, and Table S1 containing: sheet_Amyloid_3groups.csv, sheet_Ttau_3groups.csv and sheet_Ptau_3groups.csv.

## Appendix A. Meta-Analyses of Three Cerebrospinal Fluid Markers in Stable_MCI, MCI_AD, and HC

### Appendix A.1. Software and Methodology

#### Appendix A.1.1. Required Software

The analysis is performed using the open source freely available software R, a language and environment for statistics computing, version 4.1.1 (10 August 2021), R core team (2021), R Foundation for Statistical Computing, Vienna, Austria, and the ‘meta’ package (version 4.20-2 (14 December 2021) by Guido Schwarzer, University of Freiburg, Germany), which supports the book ‘Meta-Analysis with R’ by Schwarzer, Carpenter, and Rücker, Springer, Cham, 2015, first edition [63].

#### Appendix A.1.2. Meta-Analysis Approach

We use the methods detailed in Part II Chapter 2 and Part III Chapter 5.1. of ‘Meta-Analysis with R’ by Schwarzer, Carpenter, and Rücker, Springer, Cham, first edition, which follows the Cochrane Handbook for Systematic Reviews of Interventions [64].

##### Effect Measure

The analysis focusses on two comparisons: Stable_MCI (non-progressive mild cognitive impairment throughout the period of clinical follow-up) compared with MCI_AD patients (those with MCI who will progress to a diagnosis of Alzheimer’s disease during clinical follow-up), and MCI_AD compared with HC (healthy control). All measurements of interest are continuous. To accommodate different measurement technologies or analytical protocols potentially resulting in incompatible scales across the studies, a standardized mean difference is chosen to measure the effect. We use Hedges’ g, which is based on pooled sample variance and very similar to Cohen’s d but more appropriate to the group sizes in the present analysis.

##### Models and Significance Tests for Overall Effects

We conduct the meta-analyses using both a fixed effects model and a random effects model. The fixed effects model assumes that the individual studies included in the meta-analysis are sampled from the same population, so their observed means are the effect size, give or take an error term. To accommodate for differences in precision, weights inverse to the individual studies’ variances are used in the construction of the overall effect estimator. The random effects model assumes that the individual studies’ effects are normally distributed with variance tau^2^. While the fixed effects model attributes differences between observed effects entirely to sampling error, the random effects model attributes some of them to true differences between effect sizes across the studies. Significance tests for the overall effects are based on inverse variance methods.

##### Measurements and Tests of Between-Study Heterogeneity

In the context of this study, due to inter-lab differences, systematic differences between the study populations, and changes in diagnostic standards over time, a random effects model seems the right choice. To confirm this, we estimate measures of heterogeneity, the weighted sum of squares about the fixed effect estimate Q, and related quantities I^2^ and H. Finally, we perform a heterogeneity test using that Q has a Chi^2^ distribution under the Null tau^2^ = 0. Higgins et al. [65] suggest guidelines for the interpretation of I^2^ classifying 25% as low, 50% as medium, and 75% as high heterogeneity. While high heterogeneity indicates dissimilarity between the individual studies, the meta-analysis can still be valid. The size of the individual studies is of relevance here, as smaller studies have higher variation and, given they have been published, bear the risk of overestimating the effect size. While a fixed effects model tends to assign relatively lower weights to smaller studies due to their higher variability, a random effects model will weigh the studies more equally. It has been recommended by Israel et al. [66] to take into account the clinical context and to compare the results of fixed and random effects model analyses.

##### Normality Assumptions

By default, confidence intervals for the individual studies’ are calculated from means and SDs using a normal assumption. By Cochrane’s handbook (Section 10.5.3 in [64]), Altman’s rule of thumb can be used to check normality based on a lower bound in conjunction with mean and SD.

Confidence interval and *p*-value for a significance test of the overall effect size in a meta-analysis are also based on a normality assumption. Given the moderate number of studies, formal inference about normality including checks for heavy tails is not possible, but visual inspection of the distributions shows approximate normality.

##### Bias Analysis

Funnel plots are used to visualize the relationship between the effect size and a measure of its precision, here the standard error. They can give evidence of bias, in particular publication bias. Filled circles represent estimated effect sizes and their precision (standard error) for each individual study. Also shown are fixed effect estimates (vertical dashed line) with 95% confidence interval limits (diagonal dashed lines) and the random effects estimate (vertical dotted line). Smaller studies (with larger standard errors) are expected to scatter more than larger studies, giving the funnel plot’s characteristic triangular-shaped scatter. Publication bias filtering out studies that do not show the desired result tend to lead to an asymmetric plot. Typically, it is smaller studies that are not included leaving the lower right or the lower left area of the plot empty.

##### Summary Plot of the Meta-Analysis Results

Forest plots provide a graphical and numerical summary of the results of a meta-analysis and have become a common part of the Cochrane review framework. They list the individual studies with their sample sizes, study means, and SDs, and the meta-analysis’ effect size with confidence interval, both numerically and visually, together with the random and fixed effect model estimates. Heterogeneity measures are also included.

### Appendix A.2. Amyloid

Summary measures for amyloid (concentration of Aβ1-42 in CSF) from all studies are saved in the Supplementary Materials file sheet_Amyloid_3groups.csv for all conditions (stable MCI (MCI_St), MCI progressing to Alzheimer’s disease (MCI_AD), and healthy control (HC)). After reading the data, we conduct separate meta-analyses comparing MCI_AD to each of the other two conditions.

data <- **read.csv**(“sheet_Amyloid_3groups.csv”, as.is=TRUE)

#### Appendix A.2.1. Stable MCI (MCI_St) versus MCI_AD

##### Models and Significance Tests for Overall Effect and Between-Study Heterogeneity

meta <- **metacont**(N.MCI_St, Mean.MCI_St, SD.MCI_St, N.MCI_AD, Mean.MCI_AD, SD.MCI_AD,
sm=“SMD”, data=data, studlab=**paste**(Author, Year))
meta$label.e <- “MCI_St”
meta$label.c <- “MCI_AD”
**print**(meta, digits=2)
## SMD 95%-CI %W(fixed) %W(random)
## Bjerke 2009 1.70 [ 1.11; 2.28] 2.1 4.3
## Mattsson 2009 0.54 [ 0.38; 0.69] 30.4 5.7
## Hertze 2010 0.90 [ 0.53; 1.26] 5.5 5.1
## Palmqvist 2012 1.46 [ 1.04; 1.87] 4.2 4.9
## Spencer 2019 0.76 [ 0.45; 1.06] 8.0 5.3
## Hansson 2006 1.50 [ 1.08; 1.92] 4.2 4.9
## Hansson 2007 1.03 [ 0.63; 1.43] 4.6 5.0
## Hampel 2004 0.79 [ 0.22; 1.36] 2.3 4.3
## Herukka 2007 0.85 [ 0.38; 1.32] 3.4 4.7
## Lanari 2009 1.30 [ 0.66; 1.94] 1.8 4.1
## Papaliagkas 2009 1.09 [ 0.15; 2.04] 0.8 3.0
## Eckerstrom 2010 1.26 [ 0.50; 2.02] 1.3 3.6
## Kester 2011 0.93 [ 0.51; 1.35] 4.2 4.9
## Seppala 2011 0.81 [ 0.21; 1.41] 2.0 4.2
## Buchhave 2012 1.93 [ 1.47; 2.39] 3.4 4.8
## Parnetti 2012 3.46 [ 2.79; 4.14] 1.6 3.9
## Prestia 2013 1.14 [ 0.49; 1.79] 1.7 4.0
## Leuzy 2015 0.70 [-0.03; 1.44] 1.4 3.7
## Baldeiras 2018 1.21 [ 0.85; 1.56] 5.8 5.2
## Khoonsari 2019 1.64 [ 1.07; 2.21] 2.2 4.3
## Santangelo 2020 0.78 [ 0.32; 1.25] 3.4 4.7
## Eckerstrom 2020 0.94 [ 0.58; 1.30] 5.7 5.2
##
## Number of studies combined: k = 22
## Number of observations: o = 2581
##
## SMD 95%-CI z p-value
## Fixed effect model 0.97 [0.88; 1.05] 22.18 < 0.0001
## Random effects model 1.19 [0.96; 1.42] 10.10 < 0.0001
##
## Quantifying heterogeneity:
## tau^2 = 0.2360; tau = 0.4858; I^2 = 83.8% [76.6%; 88.8%]; H = 2.48 [2.07; 2.98]
##
## Test of heterogeneity:
## Q d.f. p-value
## 129.49 21 < 0.0001
## ##Details on meta-analytical method:
## - Inverse variance method
## - DerSimonian-Laird estimator for tau^2
## - Hedges’ g (bias corrected standardised mean difference)

Estimates of the measures of heterogeneity (tau^2 = 0.2360, I^2 = 83.8% [76.6%; 88.8%], H = 2.48 [2.07; 2.98]) and the test for heterogeneity (Q = 129.49, *p* < 0.0001) indicate high statistical heterogeneity across the individual studies. Despite this, both fixed effect and random effects meta-analysis show a statistically significant effect (0.97 [0.88; 1.05], *p* < 0.0001, and 1.19 [0.96; 1.42], *p* < 0.0001, respectively) in the standardized mean difference.

We note very unbalanced weights in the fixed effect model assigning study 2 (Mattsson 2009) a weight of 30.4%, while all others have at most 8%. As discussed in Appendix A.1.2, this is expected due to the much larger size of study 2 as compared to the other studies in this analysis. The random effects model still assigns the highest weight to study 2, but with 5.7% it is only slightly higher than the other studies weights ranging from 3.0% to 5.3%. Still having the highest weight, it is only 6.6% followed by 10 other studies weighing at least 5%. While study 2′s effect size is on the lowest end of the spectrum, it is not an outlier and the random effect model’s estimate is very similar to the fixed effect’s model estimate, providing evidence for the validity of the meta-analysis despite the heterogeneity.

##### Normality Assumptions

The distribution of the effect sizes for stable MCI (MCI_St) versus MCI_AD is calculated as follows and shown in Figure A1:

**hist**(meta$TE, xlim=c(0,4), breaks=12, cex.main=0.9, main=“Histogram of standardised
mean difference”, xlab=“Standardised mean difference (SMD)”)

##### Bias Analysis

The funnel plot is calculated as follows and shown in Figure A2:

	      **funnel**(meta)

##### Summary Plot of the Meta-Analysis Results

See forest plot file, Figure 7 in the main manuscript, plotted as follows:

**pdf**(file=“forest_Amyloid_MCI_StvsMCI_AD.pdf”, paper=“a4r”)
**forest**(meta, xlab=“Standardised difference in mean response (MCI_St - MCI_AD)”,
xlab.pos=-20, smlab.pos=-20, fontsize =5)
**dev.off**()

#### Appendix A.2.2. HC versus MCI_AD

##### Models and Significance Tests for Overall Effect and Between-Study Heterogeneity

meta <- **metacont**(N.HC, Mean.HC, SD.HC, N.MCI_AD, Mean.MCI_AD, SD.MCI_AD, sm=“SMD”,
data=data, studlab=**paste**(Author, Year))

## Warning in metacont(N.HC, Mean.HC, SD.HC, N.MCI_AD, Mean.MCI_AD, SD.MCI_AD, :
## Note, studies with non-positive values for n.e and / or n.c get no weight in
## meta-analysis.

meta$label.e <- “HC”
meta$label.c <- “MCI_AD”
**print**(meta, digits=2)

## SMD 95%-CI %W(fixed) %W(random)
## Bjerke 2009 3.40 [2.63; 4.16] 2.5 6.9
## Mattsson 2009 1.31 [1.13; 1.49] 44.8 10.4
## Hertze 2010 1.54 [1.06; 2.01] 6.4 8.9
## Palmqvist 2012 NA 0.0 0.0
## Spencer 2019 NA 0.0 0.0
## Hansson 2006 2.68 [2.12; 3.25] 4.6 8.3
## Hansson 2007 1.66 [1.18; 2.15] 6.1 8.8
## Hampel 2004 1.95 [1.10; 2.80] 2.0 6.4
## Herukka 2007 1.43 [0.96; 1.90] 6.5 8.9
## Lanari 2009 NA 0.0 0.0
## Papaliagkas 2009 NA 0.0 0.0
## Eckerstrom 2010 0.77 [0.16; 1.38] 3.9 7.9
## Kester 2011 NA 0.0 0.0
## Seppala 2011 1.03 [0.31; 1.75] 2.8 7.2
## Buchhave 2012 2.47 [1.96; 2.98] 5.6 8.6
## Parnetti 2012 NA 0.0 0.0
## Prestia 2013 NA 0.0 0.0
## Leuzy 2015 NA 0.0 0.0
## Baldeiras 2018 1.36 [0.99; 1.74] 10.4 9.5
## Khoonsari 2019 1.51 [0.93; 2.09] 4.3 8.2
## Santangelo 2020 NA 0.0 0.0
## Eckerstrom 2020 NA 0.0 0.0
##
## Number of studies combined: k = 12
##
Number of observations: o = 1855
##
## SMD 95%-CI z p-value
## Fixed effect model 1.53 [1.41; 1.65] 24.86 < 0.0001
## Random effects model 1.73 [1.39; 2.07] 9.98 < 0.0001
##
## Quantifying heterogeneity:
## tau^2 = 0.2799; tau = 0.5291; I^2 = 83.7% [72.9%; 90.1%]; H = 2.47 [1.92; 3.19]
##
## Test of heterogeneity:
## Q d.f. p-value
## 67.35 11 < 0.0001
##
## Details on meta-analytical method:
## - Inverse variance method
## - DerSimonian-Laird estimator for tau^2
## - Hedges’ g (bias corrected standardised mean difference)

Estimates of the measures of heterogeneity (tau^2 = 0.2799, I^2 = 83.7% [72.9%; 90.1%], H = 2.47 [1.92; 3.19]) and the test for heterogeneity (Q = 67.35, *p* < 0.0001) indicate large statistical heterogeneity across individual studies. Despite this, both fixed effect and random effects meta-analysis show a statistically significant effect (1.53 [1.41; 1.65], *p* < 0.0001, and 1.73 [1.39; 2.07], *p* < 0.0001, respectively) in the standardized mean difference.

We note very unbalanced weights in the fixed effect model, with study 2 (Mattsson 2009) having a weight of 44.8% assigned, while all others are all under 11%. However, the random effects model gives more similar weights to the studies. The effect of study 2 is on the lower end of the spectrum, but not an outlier, and the effect size estimates of the fixed and the random effect models are quite close together, so we can argue as in Appendix A.2.1 that the heterogeneity does not undermine the meta-analysis approach.

##### Normality Assumptions

The distribution of the effect sizes for HC versus MCI_AD is calculated as follows and shown in Figure A3:

**hist**(meta$TE, xlim=c(0,4), breaks=12, cex.main=0.9, main=“Histogram of standardised mean difference”, xlab=“Standardised mean difference (SMD)”)

##### Bias Analysis

The funnel plot is calculated as follows and shown in Figure A4:

	     **funnel**  
        (meta)

##### Summary Plot of the Meta-Analysis Results

See forest plot file, Figure 8 in the main manuscript, calculated as follows:

**pdf**(file=“forest_Amyloid_HCvsMCI_AD.pdf”, paper=“a4r”)
**forest**(meta, xlab=“Standardised difference in mean response (HC - MCI_AD)”,
xlab.pos=-20, smlab.pos=-20, fontsize = 5)
**dev.off**()

### Appendix A.3. T-Tau

Summary measures for T-tau (concentration of T-tau in CSF) from all studies are saved in the Supplementary Materials file sheet_Ttau_3groups.csv for all conditions (stable MCI (MCI_St), MCI_AD, HC). After reading the data, we conduct separate meta-analyses comparing MCI_AD to each of the other two conditions.

data <- **read.csv**(“sheet_Ttau_3groups.csv”, as.is=TRUE)

#### Appendix A.3.1. Stable MCI (MCI_St) versus MCI_AD

##### Models and Significance Tests for Overall Effect and Between-Study Heterogeneity

meta <- **metacont**(N.MCI_St, Mean.MCI_St, SD.MCI_St, N.MCI_AD, Mean.MCI_AD, SD.MCI_AD,
sm=“SMD”, data=data, studlab=**paste**(Author, Year))
## Warning in metacont(N.MCI_St,
Mean.MCI_St, SD.MCI_St, N.MCI_AD, Mean.MCI_AD, :
## Note, studies with non-positive values for n.e and / or n.c get no weight in
## meta-analysis.

meta$label.e <- “MCI_St”
meta$label.c <- “MCI_AD”
**print**(meta, digits=2)
## SMD 95%-CI %W(fixed) %W(random) ## Bjerke 2009 -0.74 [-1.27; -0.21] 2.8 4.8
## Mattsson 2009 -0.95 [-1.11; -0.79] 30.3 6.6
## Hertze 2010 -1.09 [-1.46; -0.72] 5.7 5.6
## Palmqvist 2012 -1.08 [-1.48; -0.69] 5.0 5.5
## Spencer 2019 -0.32 [-0.62; -0.03] 9.0 6.0
## Hansson 2006 -1.40 [-1.81; -0.99] 4.6 5.4
## Hansson 2007 NA 0.0 0.0
## Hampel 2004 -0.29 [-0.84; 0.26] 2.6 4.7
## Herukka 2007 -1.19 [-1.67; -0.70] 3.3 5.0
## Lanari 2009 -0.62 [-1.21; -0.02] 2.2 4.4
## Papaliagkas 2009 NA 0.0 0.0
## Eckerstrom 2010 -1.14 [-1.89; -0.39] 1.4 3.7
## Kester 2011 -1.12 [-1.54; -0.69] 4.3 5.3
## Seppala 2011 -1.19 [-1.82; -0.56] 2.0 4.2
## Buchhave 2012 -1.22 [-1.64; -0.81] 4.5 5.4
## Parnetti 2012 -2.99 [-3.61; -2.37] 2.0 4.3
## Prestia 2013 NA 0.0 0.0
## Leuzy 2015 -0.40 [-1.12; 0.31] 1.5 3.8
## Baldeiras 2018 -1.07 [-1.42; -0.72] 6.4 5.8
## Khoonsari 2019 -1.85 [-2.44; -1.26] 2.2 4.4
## Santangelo 2020 -0.64 [-1.10; -0.18] 3.7 5.2
## Eckerstrom 2020 -0.64 [-1.10; -0.18] 3.7 5.2
## Brys 2009 -1.00 [-1.55; -0.46] 2.7 4.7
##
## Number of studies combined: k = 20
## Number of observations: o = 2344
##
## SMD 95%-CI z p-value
## Fixed effect model -0.97 [-1.06; -0.89] -21.56 < 0.0001
## Random effects model -1.03 [-1.24; -0.82] -9.66 < 0.0001
##
## Quantifying heterogeneity:
## tau^2 = 0.1665; tau = 0.4081; I^2 = 79.0% [68.2%; 86.1%]; H = 2.18 [1.77; 2.69]
##
## Test of heterogeneity:
## Q d.f. p-value
## 90.45 19 < 0.0001
##
## Details on meta-analytical method:
## - Inverse variance method
## - DerSimonian-Laird estimator for tau^2
## - Hedges’ g (bias corrected standardised mean difference)

Estimates of the measures of heterogeneity (tau^2 = 0.1665, I^2 = 79.0% [68.2%; 86.1%], H = 2.18 [1.77; 2.69]) and the test for heterogeneity (Q = 90.45, *p* < 0.0001) indicate high statistical heterogeneity across the individual studies. Despite this, both fixed effect and random effects meta-analysis show statistically significant effects (−0.97 [−1.06; −0.89], *p* < 0.0001, and −1.03 [−1.24; −0.82], *p* < 0.0001, respectively) in the standardized mean difference.

As in the other comparisons, study 2 has a much higher weight (30.3%) than any of the other ones (at most 9%). However, the random effects model assigns the weights more similarly. The effect of study 2 is within the range of the individual studies and the effect size estimates of the fixed and the random effect models are nearly the same, so we can argue, as in Appendix A.2.1, that the heterogeneity does not undermine the meta-analysis approach.

##### Normality Assumptions

The distribution of the effect sizes for stable MCI versus MCI_AD is calculated as follows and shown in Figure A5:

**hist**(**as.numeric**(meta$TE, na.rm=TRUE), xlim=c(-3,0), breaks=12, cex.main=0.9, main=“H
istogram of standardised mean difference”, xlab=“Standardised mean difference (SMD)”
)

##### Bias Analysis

The funnel plot is calculated as follows and shown in Figure A6:

	      **funnel**  
        (meta)

##### Summary Plot of the Meta-Analysis Results

See forest plot file, Figure 9 in the main manuscript, calculated as follows:

**pdf**(file=“forest_Ttau_MCI_StvsMCI_AD.pdf”, paper=“a4r”)
forest(meta, xlab=“Standardised difference in mean response (MCI_St - MCI_AD)”,
xlab.pos=-20, smlab.pos=-20, fontsize =5)
**dev.off**()

#### Appendix A.3.2. HC versus MCI_AD

##### Models and Significance Tests for Overall Effect and Between-Study Heterogeneity

meta <- **metacont**(N.HC, Mean.HC, SD.HC, N.MCI_AD,
Mean.MCI_AD, SD.MCI_AD, sm=“SMD”, data=data, studlab=**paste**(Author, Year))

## Warning in metacont(N.HC, Mean.HC, SD.HC, N.MCI_AD, Mean.MCI_AD, SD.MCI_AD, :
## Note, studies with non-positive values for n.e and / or n.c get no weight in
## meta-analysis.

meta$label.e <- “HC”
meta$label.c <- “MCI_AD”
**print**(meta, digits=2)

## SMD 95%-CI %W(fixed) %W(random)
## Bjerke 2009 -0.60 [-1.13; -0.08] 5.4 9.0
## Mattsson 2009 -1.17 [-1.34; -0.99] 47.3 19.2
## Hertze 2010 -0.85 [-1.29; -0.41] 7.8 11.0
## Palmqvist 2012 NA 0.0 0.0
## Spencer 2019 NA 0.0 0.0
## Hansson 2006 NA 0.0 0.0
## Hansson 2007 NA 0.0 0.0
## Hampel 2004 -1.92 [-2.77; -1.07] 2.1 4.6
## Herukka 2007 -1.31 [-1.77; -0.84] 6.8 10.3
## Lanari 2009 NA 0.0 0.0
## Papaliagkas 2009 NA 0.0 0.0
## Eckerstrom 2010 -1.81 [-2.48; -1.14] 3.3 6.5
## Kester 2011 NA 0.0 0.0
## Seppala 2011 -0.76 [-1.47; -0.06] 3.0 6.1
## Buchhave 2012 -1.15 [-1.57; -0.73] 8.5 11.5
## Parnetti 2012 NA 0.0 0.0
## Prestia 2013 NA 0.0 0.0
## Leuzy 2015 NA 0.0 0.0
## Baldeiras 2018 -1.36 [-1.74; -0.99] 10.6 12.8
## Khoonsari 2019 -0.72 [-1.26; -0.19] 5.2 8.8
## Santangelo 2020 NA 0.0 0.0
## Eckerstrom 2020 NA 0.0 0.0
## Brys 2009 NA 0.0 0.0
##
## Number of studies combined: k = 10
## Number of observations: o = 1758
##
## SMD 95%-CI z p-value
## Fixed effect model -1.14 [-1.26; -1.02] -18.35 < 0.0001
## Random effects model -1.13 [-1.33; -0.93] -10.88 < 0.0001
##
## Quantifying heterogeneity:
## tau^2 = 0.0481; tau = 0.2192; I^2 = 50.5% [0.0%; 76.0%]; H = 1.42
[1.00; 2.04]
##
## Test of heterogeneity:
## Q d.f. p-value
## 18.18 9 0.0331
##
## Details on meta-analytical method:
## - Inverse variance method
## - DerSimonian-Laird estimator for tau^2
## - Hedges’ g (bias corrected standardised mean difference)

Estimates of the measures of heterogeneity (tau^2 = 0.0481, I^2 = 50.5% [0.0%; 76.0%], H = 1.42 [1.00; 2.04]) and the test for heterogeneity (Q = 18.18, *p* = 0.0331) indicate large statistical heterogeneity across the individual studies. Despite this, both fixed effect and random effects meta-analysis show statistically significant effects (−1.14 [−1.26; −1.02], *p* < 0.0001, and −1.13 [−1.33; −0.93], *p* < 0.0001, respectively) in the standardized mean difference.

We note very unbalanced weights in the fixed effect model with study 2 (Mattsson 2009) having a weight of 47.3% assigned, while all others are under 11%. The random effects model gives more similar weights to all studies.

As in the other comparisons, study 2 has a much higher weight (47.3%) while all other ones are below 11%. However, the random effects model assigns the weights more similarly. The effect of study 2 is within the range of the individual studies and the effect size estimates of the fixed and the random effect models are nearly the same, so we can argue as in Appendix A.2.1 that the heterogeneity does not undermine the meta-analysis approach.

##### Normality Assumptions

The distribution of the effect sizes for HC versus MCI_AD is calculated as follows and shown in Figure A7:

**hist**(**as.numeric**(meta$TE, na.rm=TRUE), xlim=c(-3,0), breaks=12, cex.main=0.9, main=“H
istogram of standardised mean difference”, xlab=“Standardised mean difference (SMD)”
)

##### Bias Analysis

The funnel plot is calculated as follows and shown in Figure A8:

	      **funnel**  
        (meta)

##### Summary Plot of the Meta-Analysis Results

See forest plot file, Figure 10 in the main manuscript, calculated as follows:

**pdf**(file=“forest_Ttau_HCvsMCI_AD.pdf”, paper=“a4r”)
forest(meta, xlab=“Standardised difference in mean response (HC - MCI_AD)”,
xlab.pos=-20, smlab.pos=-20, fontsize = 5)
**dev.off**()

### Appendix A.4. P-Tau

Summary measures for P-tau (concentration of P-tau in CSF) from all studies are saved in the Supplementary Materials file sheet_Ptau_3groups.csv for all conditions (stable MCI (MCI_St), MCI_AD, HC). After reading the data, we conduct separate meta-analyses comparing MCI_AD to each of the other two conditions.

data <- **read.csv**(“sheet_Ptau_3groups.csv”, as.is=TRUE)

#### Appendix A.4.1. Stable MCI (MCI_St) versus MCI_AD

##### Models and Significance Tests for Overall Effect and Between-Study Heterogeneity

meta <- **metacont**(N.MCI_St, Mean.MCI_St, SD.MCI_St, N.MCI_AD, Mean.MCI_AD, SD.MCI_AD,
sm=“SMD”, data=data, studlab=**paste**(Author, Year))

## Warning in metacont(N.MCI_St, Mean.MCI_St, SD.MCI_St, N.MCI_AD, Mean.MCI_AD,:
## Note, studies with non-positive values for n.e and / or n.c get no weight in
## meta-analysis.

meta$label.e <- “MCI_St”
meta$label.c <- “MCI_AD”
print(meta, digits=2)

## SMD 95%-CI %W(fixed) %W(random)
## Bjerke 2009 -5.68 [-6.76; -4.60] 0.7 3.8
## Mattsson 2009 -0.97 [-1.14; -0.81] 29.1 4.9
## Hertze 2010 -1.07 [-1.44; -0.70] 5.5 4.8
## Palmqvist 2012 -0.97 [-1.36; -0.58] 5.0 4.8
## Spencer 2019 -0.60 [-0.90; -0.30] 8.4 4.8
## Hansson 2006 -1.40 [-1.81; -0.98] 4.4 4.7
## Hansson 2007 1.03 [ 0.63; 1.43] 4.8 4.8
## Hampel 2004 0.79 [ 0.22; 1.36] 2.3 4.6
## Herukka 2007 -1.05 [-1.53; -0.57] 3.3 4.7
## Lanari 2009 -2.39 [-3.16; -1.63] 1.3 4.3
## Papaliagkas 2009 1.09 [ 0.15; 2.04] 0.8 4.0
## Eckerstrom 2010 1.26 [ 0.50; 2.02] 1.3 4.3
## Kester 2011 0.93 [ 0.51; 1.35] 4.3 4.7
## Seppala 2011 -0.59 [-1.18; 0.00] 2.2 4.6
## Buchhave 2012 -1.07 [-1.48; -0.66] 4.5 4.8
## Parnetti 2012 -7.97 [-9.23; -6.70] 0.5 3.5
## Prestia 2013 NA 0.0 0.0
## Leuzy 2015 -0.15 [-0.86; 0.56] 1.5 4.4
## Baldeiras 2018 -0.96 [-1.30; -0.61] 6.3 4.8
## Khoonsari 2019 -1.56 [-2.13; -1.00] 2.3 4.6
## Santangelo 2020 -1.03 [-1.51; -0.55] 3.3 4.7
## Eckerstrom 2020 -1.22 [-1.59; -0.85] 5.6 4.8
## Brys 2009 -1.19 [-1.74; -0.63] 2.5 4.6
##
## Number of studies combined: k = 22
## Number of observations: o = 2603
##
## SMD 95%-CI z p-value
## Fixed effect model -0.80 [-0.89; -0.72] -18.08 < 0.0001
## Random effects model -1.03 [-1.47; -0.59] -4.56 < 0.0001
##
## Quantifying heterogeneity:
## tau^2 = 1.0197; tau = 1.0098; I^2 = 95.6% [94.4%; 96.6%]; H = 4.77 [4.21; 5.40]
##
## Test of heterogeneity:
## Q d.f. p-value
## 477.66 21 < 0.0001
##
## Details on meta-analytical method:
## - Inverse variance method
## - DerSimonian-Laird estimator for tau^2
## - Hedges’ g (bias corrected standardised mean difference)

Estimates of the measures of heterogeneity (tau^2 = 1.0197, I^2 = 95.6% [94.4%; 96.6%], H = 4.77 [4.21; 5.40]) and the test for heterogeneity (Q = 477.66, *p* < 0.0001) indicate that very large statistical heterogeneity across the individual studies is present. Despite this, both fixed effect and random effects meta-analysis show statistically significant effects (−0.80 [−0.89; −0.72], *p* < 0.0001, and −1.03 [−1.47; −0.59], *p* < 0.0001, respectively) in the standardised mean difference.

As in the other comparisons, study 2 has a much higher weight (29.1%) while all other ones are below 9%. However, the random effects model assigns the weights more similarly. The effect of study 2 is within the range of the individual studies and the effect size estimates of the fixed and the random effect models are still not that far apart, so we can argue as in Appendix A.2.1 that the heterogeneity does not undermine the meta-analysis approach.

##### Normality Assumptions

The distribution of the effect sizes for stable MCI (MCI_St) versus MCI_AD is calculated as follows and shown in Figure A9:

**hist**(**as.numeric**(meta$TE, na.rm=TRUE), xlim=c(-10,4), breaks=12, cex.main=0.9, main=“
Histogram of standardised mean difference”, xlab=“Standardised mean difference (SMD)
” )

##### Bias Analysis

The funnel plot is calculated as follows and shown in Figure A10:

	      **funnel**  
        (meta)

##### Summary Plot of the Meta-Analysis Results

See separately saved forest plot file, Figure 11 in the main manuscript, plotted as follows:

**pdf**(file=“forest_Ptau_MCI_StvsMCI_AD.pdf”, paper=“a4r”)
**forest**(meta, xlab=“Standardised difference in mean response (MCI_St - MCI_AD)”,
xlab.pos=-20, smlab.pos=-20, fontsize =5)
**dev.off**()

#### Appendix A.4.2. HC vs. MCI_AD

##### Models and Significance Tests for Overall Effect and Between-Study Heterogeneity

meta <- **metacont**(N.HC, Mean.HC, SD.HC, N.MCI_AD, Mean.MCI_AD, SD.MCI_AD,
sm=“SMD”, data=data, studlab=**paste**(Author, Year))

## Warning in metacont(N.HC, Mean.HC, SD.HC, N.MCI_AD, Mean.MCI_AD, SD.MCI_AD, :
## Note, studies with non-positive values for n.e and / or n.c get no weight in
## meta-analysis.

meta$label.e <- “HC”
meta$label.c <- “MCI_AD”
**print**(meta, digits=2)

## SMD 95%-CI %W(fixed) %W(random)
## Bjerke 2009 NA 0.0 0.0
## Mattsson 2009 -1.13 [-1.31; -0.96] 55.9 55.9
## Hertze 2010 -0.99 [-1.43; -0.55] 8.9 8.9
## Palmqvist 2012 NA 0.0 0.0
## Spencer 2019 NA 0.0 0.0
## Hansson 2006 NA 0.0 0.0
## Hansson 2007 NA 0.0 0.0
## Hampel 2004 NA 0.0 0.0
## Herukka 2007 -1.14 [-1.59; -0.68] 8.4 8.4
## Lanari 2009 NA 0.0 0.0
## Papaliagkas 2009 NA 0.0 0.0
## Eckerstrom 2010 NA 0.0 0.0
## Kester 2011 NA 0.0 0.0
## Seppala 2011 -0.47 [-1.15; 0.22] 3.7 3.7
## Buchhave 2012 -1.04 [-1.46; -0.63] 10.2 10.2
## Parnetti 2012 NA 0.0 0.0
## Prestia 2013 NA 0.0 0.0
## Leuzy 2015 NA 0.0 0.0
## Baldeiras 2018 -1.21 [-1.57; -0.84] 13.0 13.0
## Khoonsari 2019 NA 0.0 0.0
## Santangelo 2020 NA 0.0 0.0
## Eckerstrom 2020 NA 0.0 0.0
## Brys 2009 NA 0.0 0.0
##
## Number of studies combined: k = 6
## Number of observations: o = 1749
##
## SMD 95%-CI z p-value
## Fixed effect model -1.10 [-1.23; -0.96] -16.29 < 0.0001
## Random effects model -1.10 [-1.23; -0.96] -16.29 < 0.0001
##
## Quantifying heterogeneity:
## tau^2 = 0; tau = 0; I^2 = 0.0% [0.0%; 74.6%]; H = 1.00 [1.00; 1.99]
##
## Test of heterogeneity:
## Q d.f. p-value
## 4.06 5 0.5414
##
## Details on meta-analytical method:
## - Inverse variance method
## - DerSimonian-Laird estimator for tau^2
## - Hedges’ g (bias corrected standardised mean difference)

The test for heterogeneity is negative, and the fixed effect and random effects models show the same statistically significant effect (−1.10 [−1.23; −0.96], *p* < 0.0001) in the standardized mean difference.

We note very unbalanced weights in both fixed effect and random effects model with study 2 dominating with a weight of 55.9%, while all others are no more than 13%. While less extreme, such heterogeneity was also observed in the other comparisons. In this case, the random effects model does not make a difference. The effects in the individual studies not being too dissimilar adds some robustness to the meta-analysis approach. There are, however, some limitations due to the large number of missing values, as this comparison was only included in some of the individual studies.

##### Normality Assumptions

The distribution of the effect sizes for HC versus MCI_AD is calculated as follows and shown in Figure A11:

**hist**(**as.numeric**(meta$TE, na.rm=TRUE), xlim=c(-1.5,0), breaks=12, cex.main=0.9, main=
“Histogram of standardised mean difference”, xlab=“Standardised mean difference (SMD
)”)

##### Bias Analysis

The funnel plot is calculated as follows and shown in Figure A12:

	      **funnel**(meta)

##### Summary Plot of the Meta-Analysis Results

See forest plot file, Figure 12 in the main manuscript, calculated as follows:

**pdf**(file=“forest_Ptau_HCvsMCI_AD.pdf”, paper=“a4r”)
forest(meta, xlab=“Standardised difference in mean response (HC - MCI_AD)”,
xlab.pos=-20, smlab.pos=-20, fontsize =5)
**dev.off**()
